# NMR Spectroscopy in Diagnosis and Monitoring of Methylmalonic and Propionic Acidemias

**DOI:** 10.3390/biom14050528

**Published:** 2024-04-28

**Authors:** Calin Deleanu, Alina Nicolescu

**Affiliations:** 1 “Costin D. Nenitescu” Institute of Organic and Supramolecular Chemistry, Spl. Independentei 202-B, RO-060023 Bucharest, Romania; 2 “Petru Poni” Institute of Macromolecular Chemistry, Aleea Grigore Ghica Voda 41-A, RO-700487 Iasi, Romania

**Keywords:** methylmalonic acidemia, propionic acidemia, B_12_ deficiency, nuclear magnetic resonance, NMR, MRS, MRI

## Abstract

Although both localized nuclear magnetic resonance spectroscopy (MRS) and non-localized nuclear magnetic resonance spectroscopy (NMR) generate the same information, i.e., spectra generated by various groups from the structure of metabolites, they are rarely employed in the same study or by the same research group. As our review reveals, these techniques have never been applied in the same study of methylmalonic acidemia (MMA), propionic acidemia (PA) or vitamin B_12_ deficiency patients. On the other hand, MRS and NMR provide complementary information which is very valuable in the assessment of the severity of disease and efficiency of its treatment. Thus, MRS provides intracellular metabolic information from localized regions of the brain, while NMR provides extracellular metabolic information from biological fluids like urine, blood or cerebrospinal fluid. This paper presents an up-to-date review of the NMR and MRS studies reported to date for methylmalonic and propionic acidemias. Vitamin B_12_ deficiency, although in most of its cases not inherited, shares similarities in its metabolic effects with MMA and it is also covered in this review.

## 1. Introduction

Rare diseases are currently defined as diseases affecting less than 1 in 2000 persons. There are over 6000 known rare diseases, and the combined incidence of rare diseases is estimated to affect as much as 4% of the entire population [1]. Most of these diseases are due to inborn errors of metabolism (IEM) and they are also referred to as intoxication-type metabolic disorders due to the fact that deficient enzymes lead to the accumulation of toxic metabolites in the organism [2,3,4]. These diseases manifest with various subtypes dependent upon the responsible defective genes [5,6], and they exhibit life-threatening clinical episodes.

MMA and PA are rare metabolic diseases due to inborn errors in the propionate metabolism. Vitamin B_12_ deficiency can occur due to various factors, including inadequate dietary intake, malabsorption or an impaired metabolism of the vitamin B_12_. Although MMA and vitamin B_12_ deficiency share similarities in their metabolic effects, they are distinct conditions with different underlying causes. Proper diagnosis and differentiation between these conditions are essential for their appropriate management and treatment.

MMA can either be primary (due to an enzyme or cofactor deficiency) or secondary to a vitamin B_12_ deficiency. Primary MMA is due to a defect in the methylmalonyl-CoA mutase enzyme (which catalyzes the conversion of methylmalonyl-CoA to succinyl-CoA), leading to the excretion of several compounds like methylmalonic acid, propionylcarnitine and methylcitric acid in the body’s fluids. The first two cases of MMA were reported in 1967 by Oberholzer et al. [7]. Methylmalonic acid was isolated from urine by column chromatography and compared with an authentic standard for its melting points, elemental analysis and absorption spectrum. Combined MMA and homocystinuria (MMA-HC), also known as cobalamin C disease (cbIC), is the most common vitamin B_12_-inherited disease. PA is due to a defect in propionyl CoA carboxylase (which catalyzes the conversion of propionyl-CoA to methylmalonyl-CoA), leading to the excretion of several propionyl derivatives in the body’s fluids. PA was first explicitly described by Hommes et al. in 1968 [8], although it might be the case that a report by Childs et al. in 1961, designating a new acidemia as idiopathic hyperglycinemia and hyperglycinuria, could also have been PA [9]. Vitamin B_12_ (cobalamin, with its active form deoxyadenosylcobalamin) deficiency has been determined either alone or in association with other conditions like MMA, homocystinuria, ketone bodies and/or glycinuria [10,11,12]. Vitamin B_12_ deficiencies are more common during pregnancy and lactation and may be induced in breast-fed infants. A B_12_ deficiency has also been diagnosed in strict vegetarians [13,14]. Vitamin B_12_ deficiency has been known about for a long time [15,16,17] and it is one of the IEM with the highest incidence in newborns [18]. 

For the three pathologies discussed in this paper, as for most of the IEM, the prognosis of their evolution and their level of irreversible damage to various organs depend on how early the treatment is started and, thus, on how early they are diagnosed. Several guidelines for the diagnosis and management of these pathologies have been published [18,19,20,21]. 

Current diagnostic standards are based on GC/LC-MS analyses of urine (for PA metabolites like methylcitric acids, 3-hydroxypropionate, propionylglycine, tiglylglycine, 3-hydroxy-2-methylbutyrate and 2-methyl-acetoacetate and for MMA metabolites like methylmalonic acid, methylcitric acid, 3-hydroxypropionic acid and propionylcarnitine) and of blood plasma (with acylcarnitine and free carnitine being relevant for both PA and MMA) [22,23,24]. Ketoacidosis and hyperammonemia, particularly during episodes of metabolic decompensation, complicate the clinical pattern of these diseases. In categories at risk (families with an older index sibling or history of IEM), DNA tests as well as MS analyses of prenatal amniotic fluid for the relevant metabolites are also often performed. ESI-MS is used in high-throughput neonatal screenings [25,26,27,28,29].

The long-term therapy of PA and MMA is based on a low-protein high-energy diet free of propiogenic compounds. Carnitine, metronidazole and vitamin B_12_ are usually administered. In spite of their early detection, their long-term prognosis is poor. Recently, liver transplants have become popular as an alternative to diet and classical medication. Combined liver and kidney transplants have also been improving the condition of patients with end-stage renal failure. Transplants have been shown to improve the general condition of the patient, but have failed to prevent further excretion of disease markers [26]. However, early transplantation combined with the patients’ management by multidisciplinary teams have been showing improved outcomes in the medium term [4]. Recent advancements in genomic therapies, including mRNA, show promising results in phase I and II clinical trials [30]. 

In addition to targeted markers, metabolomics, which simultaneously evaluate multiple parameters, could improve both the early detection and long-term monitoring of these diseases, including their therapy/transplant efficiency. Currently, only MS and NMR spectroscopy are suitable techniques for large metabolomic screenings. As these techniques exhibit both advantages and drawbacks, they are most efficiently used in combination. Although non-localized ex vivo NMR has proven its potential in the urine-based diagnosis of metabolic diseases since mid-1980s [31,32,33,34,35], the technique is much less popular than MS. An excellent introductory handbook on the ^1^H NMR assignment of various IEM is provided by Engelke et al. [36]. The book was first published as early as 2002 and the third and current edition in 2014 [36]. An updated version would be very well received. Hajek and Dezortova [37] have given an introduction to clinical localized in vivo NMR spectroscopy (MRS), while also showing the comparative gain in spectral resolution when gradually moving from 1.5 to 9.4 Tesla equipment. Bertholdo et al. [38] gave an excellent introduction to brain MRI and MRS, including explanations of the effect of their experimental parameters. Sherry et al. [39] reviewed the brain MRS applications for several inborn and acquired metabolic diseases and Kreis et al. [40] have shown the strong age-dependent correlation between metabolite concentrations in brain and the influence of MRS parameters on the resulting spectra. 

The present paper provides a review of the NMR and MRS data available for MMA, PA and B_12_ deficiency. For the purpose of this review, high-resolution non-localized ex vivo NMR spectroscopy performed in vertical magnets with NMR tubes on body fluids is referred to as nuclear magnetic resonance spectroscopy (NMR). This type of spectroscopy provides only one NMR signal from the entire sample, with a high signal-to-noise (S/N) ratio and high resolution, allowing high-quality signal assignments and precise integral/intensity measurements. Localized in vivo NMR spectroscopy performed with imaging equipment and horizontal magnets on entire living organisms (humans or animals) is referred to as Magnetic Resonance Spectroscopy (MRS). This type of spectroscopy provides signals from only a localized part of the organism, allowing for an in vivo examination of precisely selected parts of organs and generating signals from normal versus pathological regions (e.g., tumors or brain lesions). As in localized spectroscopy, only one part of the sample gathered from the detection coil (antenna) is recorded; the S/N in MRS is much weaker than in NMR. Also, due to the fact that the sample is highly inhomogeneous, the resolution in MRS is much poorer than in NMR. Obviously, both types of spectroscopies have unique advantages as well as drawbacks, and they provide complementary information. MRS is usually recorded in combination with Nuclear Magnetic Resonance Imaging (MRI) as it is recorded using the same instrument. MRI is a well-established routinely used clinical investigation method and will not be exhaustively covered by the present review; however, MRI will be mentioned when it has been used in combination with MRS. When both MRI and MRS are used, the MRI is firstly recorded and further used to guide the voxel selection for the region where MRS should be recorded.

## 2. Non-Localized Ex Vivo Nuclear Magnetic Resonance Spectroscopy (NMR)

The pioneering works on body fluids’ analysis using NMR spectroscopy were published in the mid-1980s by the groups of J. K. Nicholson [32,33,34,35] and R. A. Iles [41,42,43,44,45]. 

The detection of methylmalonic and propionic acids in the urine of MMA and PA patients was briefly mentioned by Iles et al. at 400 MHz (9.4 Tesla), without further details, in the early stages of NMR applications in IEM [41,42]. The NMR spectra of urine from MMA and PA patients were mentioned by Iles one year later at the same magnetic field, without showing the NMR spectra [46], but this time additional information on several metabolites were presented. Thus, it was mentioned that, in these MMA patients, in addition to methylmalonic acid, 3-hydroxypropionate and propionylglycine have also been tracked. Ketotic episodes have been tracked by monitoring acetone, 3-hydroxybutyrate and 3-hydroxypropionate [46]. In the monitored PA patient, large variations in their concentrations of glycine, propionylglycine, 3-hydroxypropionate and acetate have been reported, but no concentration values for these metabolites and no spectra have been presented [46].

The diagnosis of MMA by NMR at 400 MHz was well detailed, independently, by the groups of Nicholson and Iles in the same year [33,47]. In these and previously mentioned papers, the method of choice for recording the NMR spectra of urine was the Hahn spin echo (HSE).

Iles’ group has published the most extensive series of studies on PA and MMA available to date. Iles disclosed well-resolved PA and MMA urine HSE spectra at 400 MHz [43,45]. They suggested the first employment of two types of 1D NMR spectra in the diagnosing of PA and MMA, namely HSE for assignment purposes and classical one-pulse ^1^H NMR for quantitation purposes [45]. Detailed chemical shift signal assignments for various groups of metabolites have been described [45]. Basically, this was last time HSE spectra have been mentioned in PA and MMA studies. A comparison between 250 MHz and 400 MHz instruments mentioned that creatine and creatinine cannot be distinguished at 250 MHz, but they are separated at 400 MHz [45]. The paper estimated that metabolites present in concentrations higher than 0.5 or 1.0 mM can be detected at 400 MHz and that higher-field spectrometers will improve the diagnosis of metabolic diseases [45]. In MMA patients, high concentrations of 3-hydroxybutyrate, acetone and isovalerylglycine have been reported, with their markers well identified in the NMR spectra [45]. In PA patients, more advanced identifications of high-concentration-specific markers have been reported for 3-hydroxypropionate, propionylglycine, propionylcarnitine, glycine-variable propionylglycine or 3-hydroxypropionate, and tiglylglycine [45]. Methylcitrate was first reported via NMR in PA but only visible in lyophilized extracts.

Carnitine/acylcarnitine, creatinine, creatine, glycine, methylmalonate, 3-hydroxypropionate, propionylglycine, tiglyglycine + tiglate have been identified in HSE spectra by Iles et al. [47]. They stated that the HSE fingerprint of MMA should be definitive, but that the PA fingerprint is quite variable even for the same patient [47]. In PA patients, signals from the conjugate of propionyl-CoA and propionylglycine have been identified with high intensities. Glycine was also mentioned “in large amounts” and 3-hydroxypropionate was “clearly visible” [43]. Typically, 3-hydroxypropionate and/or propionylglycine are visible, although sometimes neither of them is detectable. In contrast, high levels of glycine have been noticed in all patients and all samples [43]. In a patient with MMA with ketoacidosis, large amounts of methylmalonic acid, 3-hydroxybutyrate and creatine have been noticed. Creatine was mentioned as not being abnormal in young children (18 mo), but its role in relation to dehydration and poor renal reabsorption was not excluded [43]. In another five MMA patients, methylmalonic acid was always present, with only occasional ketosis episodes [43].

The NMR monitoring of a PA patient over 10 months and a MMA patient over 11 days has been reported by Iles et al. [44] and, for comparison, they included another four PA and four MMA patients with only a one-time analysis. This is the first example of a detailed NMR metabolomic monitoring (methylmalonic acid, 3-hydroxypropionate, propionylglycine, propionylcarnitine, glycine, propionylglycine, 3-hydroxypropionate, 3-hydroxybutirate, tiglylglycine, methylcitrate, creatine, betaine, creatinie, acetone, acetate, hippurate) of PA and MMA during treatment [44].

A step further was presented by Iles with the detailed monitoring of several metabolites in two PA and one MMA patient undergoing L-carnitine therapy [48,49]. Thus, the monitoring, in PA patients, of betaine, creatine, carnitine, propionylcarnitine and “total propionate” (i.e., 3-hydroxypropionate + propionylglycine + tiglylglcyine + propionylcarnitine) over up to 22 h after an L-carnitine administration have been presented as concentrations relative to creatinine. Similarly, for an MMA patient, propionylcarnitine, acetylcarnitine and “total propionate” (i.e., methylmalonate + propionylcarnitine + 3-hydroxypropionate) over 30 h after an L-carnitine administration have been presented as concentrations relative to creatinine [49]. Well-resolved and -assigned one-pulse ^1^H NMR spectra recorded at 400 MHz have been presented [49] and the same spectra have also been included in a later review by Iles [50].

Davies, Iles et al. [51] monitored two MMA patients and two other IEM during metabolic decompensation episodes. They reported as increased both the creatine/creatinine ratio and total daily creatine during decompensation, as well as their returning to normal during recovery. However, methylmalonic acid/creatinine did not fluctuate [51]. The same group mentioned their previous observation [44] of creatine in MMA and PA patients during metabolic decompensations. Evolutions of creatine, 3-hydroxypropionate, 3-hydroxybutyrate and methylmalonic acid, as graphs relative to creatinine, have been presented. Also, graphs relative to creatinine for creatine, 3-hydroxypropionate, propionylcamitine, acetoacetate, acetone, 3-hydroxybutyrate and methylmalonic acid have been presented during a carnitine treatment [51].

Following the previous carnitine therapy monitoring in MMA and PA patients [49], Iles’ group reported the monitoring of a metronidazole treatment in PA and MMA patients [52]. Two PA and two MMA patients were monitored by the NMR of their urine before and up to 20 days after beginning the metronidazole treatment. The total propionate in the PA patients was reported as the sum of propionylcarnitine, 3-hydroxypropionate, 2S,3S and 2S,3R methylcitrates, tiglylglycine and propionylglycine. For the MMA patients, methylmalonic acid was added to the previous calculation in order to calculate the total propionate. The study revealed that although the total propionate was significantly reduced by metronidazole, the propionylcarnitine was not decreased [52]. Well-resolved 500 MHz ^1^H NMR spectra of PA urine before and after the administration of metronidazole are presented [52].

In a review paper, Iles mentioned as an unpublished result the detection, via ^1^H NMR, of 2,3-butandiol in the urine of some PA patients, but no details or spectra have been published [50]. In the same review, good-quality 400 and 500 MHz one-pulse ^1^H NMR for control and MMA patients, reproduced from their previous papers [45,49], are presented.

Iles et al. reported, for the first time, a good correlation between NMR and GC-MS on metabolite/creatinine (Crn) ratios [45], with detailed comparative MS/NMR results presented one year later [44].

Pan et al. reported ^1^H NMR spectra at 500 MHz for 20 controls, one MMA case and five other types of IEM pathologies [53]. These NMR spectra have been discussed in comparison with desorption electrospray ionization mass spectrometry (DESI-MS). A principal component analysis (PCA) was used for separating the spectra and for assisting in identifying specific markers based on loading plots. A list including the NMR chemical shifts and MS fragments’ mass to charge ratios (*m*/*z*) for several normal and pathologic metabolites was presented. A correlation matrix chemical shift versus *m*/*z* is also presented. The authors advocate for the advantage of performing urine screening using a combined NMR and MS technique [53]. The paper recognized that it is not possible to determine concentrations from PCA outputs, but it pointed out that targeted metabolite concentrations may be obtained from the original ^1^H NMR spectra. However, no concentrations have been provided, although specific markers for MMA have been listed as methylmalonate, trimethylamine N-oxide (or betaine) and dimethylamine, together with three unidentified singlets [53].

Iles et al. reported, for the first time, the detection by NMR, in PA, of methylcitrate as a mixture of two isomers (2RS,3RS and 2RS,3SR). However, this was only visible in lyophilized extracts [45]. The failure to identify methylcitric acid in native urine was explained by its superposition with citrate, and it was thought that with higher magnetic fields its detection in native urine would be possible [45]. Krawczyk and Gradowska [54] have been unambiguously identifying these two methylcitrate isomers in the native urine of PA and MMA patients using 400 MHz NMR spectra. Their signal assignments have been assisted by H-C 2D NMR correlations using HMQC experiments. Their assignments have been compared, using previously reported chemical shifts, coupling constants, and 2D COSY, HMQC and HMBC correlations, with standard compounds [55]. A well-resolved PA urine one-pulse ^1^H NMR spectrum, with signal assignments, together with HMQC correlations, has been presented [54].

A clear demonstration of the selectively differentiating diagnosis of MMA versus PA by NMR was achieved by Kodama et al. in 1991 [56] using a 500 MHz (11.7 Tesla) instrument. Higher magnetic field and additional COSY experiments have been employed to directly quantify methylcitrate in the native urine of a PA patient, in addition to 3-hydroxy-n-valerate, propionylglycine, 3-hydroxy-n-butyrate, lactate, tiglylglycine, acetoacetate, 3-hydroxypropionate, betaine, glycine, hippurate, creatinine and propionyl carnitine [56]. Their concentrations relative to creatinine have been reported before and after an L-carnitine treatment [56]. The concentration of methylmalonate was reported before and after a treatment with L-carnitine and sodium benzoate [56]. Well-resolved 500 MHz one-pulse ^1^H NMR spectra of the urine from PA and MMA patients, with assignments of methylmalonate, glycine, creatinine, creatine and betaine, have been presented [56].

Lehnert and Hunkeler [57] have been running the urine spectra of MMA, PA and other IEM at 250 MHz (5.9 Tesla). One-pulse ^1^H NMR spectra for diagnosing various metabolic disorders have been run on samples adjusted to a pH of 2.5. They published a list of chemical shifts for the relevant metabolites (including methylmalonic acid and glycine) at this pH. They also admitted that 250 MHz could be too low a frequency for most studies, but they assumed that 400 and 500 MHz should be suitable for most cases [57]. They have also mentioned J-Resolved spectroscopy (JRES) in addition to COSY as a useful tool for the assignment of signals [57].

Nicolescu et al. [58] have monitored, by ^1^H NMR at 600 MHz (14.1 Tesla), five MMA patients for several months at up to 9-year intervals. A comprehensive set of metabolites have been monitored in their urine. It was shown that, in addition to being a therapy assessment, the levels of methylmalonic acid and glycine in urine can indicate the type of MMA mutation (MMAA versus MMUT). Average concentrations and ranges of the following MMA-associated metabolites—glycine, methylmalonic acid, orotic acid, orotidine, L-carnitine and propionylcarnitine—have been presented for all monitored patients. The concentration ranges of these metabolites in the literature data have also been presented. Ranges for another 20 non-specific metabolites have also been published for all five patients, together with control ranges from the literature data. A one-pulse ^1^H NMR spectrum with assignments, a JRES and a COSY spectrum from an MMA case are presented as examples [58].

Blasco et al. [59] have demonstrated that MMA can be efficiently diagnosed by ^1^H NMR on dry filter paper saturated with urine. They have compared, for five healthy subjects, an MMA patient and three other IEM patients, the NMR spectra of both fresh urine and reconstituted solutions from filter paper. The same solution, after being dried on filter paper and stored in different conditions for different time intervals, was reconstituted in pure D_2_O and recorded once again. They concluded that the technique is at least well suited for the controls and studied IEM, including MMA [59]. This technique could definitely improve the diagnosis and therapy monitoring of MMA, as both blood spots and urine-saturated paper could be easily shipped to more remote laboratories. Although urine on filter paper has been used in some other analyses like PCR [60], GC/LC-MS and TLC [61,62,63,64,65,66,67,68], no other groups followed this approach using NMR.

Engelke et al. [36] published, in their book, several reference ^1^H NMR spectra of urine from a PA and three types of MMA patients.

A few other urine NMR studies have dealt with PA and MMA, but they have limited diagnosis utility and they remain more historical studies. Thus, Yamaguchi et al. [69] have published a urine MMA spectrum in comparison with a normal spectrum and another IEM pathology recorded at 90 MHz (2.1 Tesla). However, only the methyl doublet of methylmalonic acid is a distinguishable feature in the published spectrum, as 90 MHz is quite a low field for complex mixtures. The published spectra have limited diagnosis value; nevertheless, the paper showed that, even with this low field, an MMA suspicion can be quickly raised by NMR [69]. A brief communication by Bamforth et al. mentioned that they have analyzed urine from 85 patients with confirmed IEM, including MMA patients ranging from 1–15 yo [70]. They mentioned 500 MHz ^1^H NMR spectra of urine. No MMA spectra were presented. They have also reported neural network classifications without further details [70]. A qualitative differentiation, based on a combined ^1^H NMR profile at 400 MHz and GC-MS, between several IEM, including PA, was explored by Pulido et al. [71]. The study, published in an era of rather usual 600 MHz biomedical approaches, aimed to confirm the usefulness of the much cheaper medium-field 400 MHz in the diagnosis of several IEs, including PA. Thus, the magnetic field employed was the one used in the early days of NMR studies of MMA and PA but recorded with more modern equipment. The study included 36 controls, 2 PA patients and samples from several other IEM which were initially diagnosed by GC-MS. For PA, the following specific metabolites were detected by NMR: propionic acid, 3-hydroxypropionic acid and propionylglycine, while the following specific metabolites have been identified by MS: 3-hydroxypropionic acid, 3-hydroxyvaleric acid, propionylglycine, tiglylglycine and methylcitric acid. An NMR spectrum from a control, together with spectra for various IEM, including one PA spectrum, is presented. A list of the NMR chemical shifts of various metabolites in the controls and specific IEM are presented, but no concentration information is given. Multivariate statistical analyses have shown some separation between healthy and IEM urine [71], but the amount of data were small and the results are not very convincing. This study [71], as do the other PA- and MMA-related statistical analyses reported up to now, remains a proof of concept with limited practicality in diagnosis, mainly due to the very small amount of data [53,70].

A limited number of studies involved biological fluids other than urine.

Using hyphenated HPLC-NMR spectroscopy, Monostory et al. [72] identified a previously unknown interference in the routine analysis of the dried blood spots (DBSs) used in inborn screening, affecting the methylmalonic acid determination of PA patients. Thus, their paper reports on the identification of five isobaric (same molecular mass) compounds in serum and urine from MMA and PA patients, which elute close to methylmalonic acid, inducing errors in the identification and quantitation of the latter. In particular, changing eluting conditions and suboptimal settings of the evaluation software have been reported to induce false-positive MMA diagnoses. These MS-interfering metabolites have been identified by NMR as 2-methyl-3-hydroxybutyrate, 3-hydroxyisovalerate, 2-hydroxyisovalerate, 3-hydroxyvalerate and succinate. A sixth compound in low abundance has also been observed, but its structure was not identified. Out of these isobaric compounds, only succinate has been previously reported to interfere with methylmalonic acid in the MS analysis of DBSs. The study used both DBSs and urine and performed several NMR experiments including ^1^H NMR, HSQC, HMBC and TOCSY, recorded at 500 and 600 MHz [72].

Commodari et al. have quantified methylmalonic acid in a B_12_-deficient patient by ^1^H NMR of their cerebrospinal fluid (CSF) at 300 MHz [73]. Lactate, alanine, acetate, glutamine, citrate, creatine + creatinine, glucose and methylmalonic acid have been identified in their CSF spectra. Several other signals were present in the NMR spectrum but they could not be assigned to particular metabolites. The methylmalonic acid concentration in the CSF of the patient was estimated to be 154 µM. The chemical shift dependency of pH on methylmalonic acid was also reported as a titration curve [73]. For signal assignment purposes, COSY spectra have been recorded for both a control and a B_12_-deficient patient [73].

A study using ^1^H NMR on cord blood revealed markers for the potential risk of MMA and other two diseases in gestational hypothyroidism (GHT), which is a frequent pregnancy-related thyroid disfunction [74].

Although the prenatal diagnosis of PA and MMA conditions via a classical analysis of amniotic fluid is possible [75], no attempt to explore the potential of NMR in this respect has been published. Until now, for these pathologies, CSF remains the only fluid explored using NMR, in addition to the well-proven urine-based diagnoses.

## 3. NMR Methodology

As it concerns the NMR methodology in terms of field strength, 400 MHz (9.4 Tesla) magnets have been employed in most of the studies related to the reviewed pathologies [33,41,42,43,44,45,46,47,49,50,51,54,55,58,71,73], and the results are suitable for the medical diagnosis of these pathologies. One 90 MHz (2.1 Tesla) study was reported [69]. However, the spectra’s quality at 90 MHz is too low for medical diagnosis. Several 250 MHz (5.9 Tesla) studies [49,50,51,57] have also been reported and, although valuable information was obtained, we consider this field still too low for confident medical diagnoses. One study at 300 MHz (7.1 Tesla) was reported on CSF [73] and, for this particular fluid, the spectra look acceptable for medical research. Several 500 MHz (11.7 Tesla) [50,52,53,56,59,70,72] and 600 MHz (14.1 Tesla) [58,72,74] studies have been reported, with high-quality spectra. In spite of the current tendency to use 600 MHz instruments for medical diagnoses, these instruments have been seldom used for the reviewed pathologies.

There is very little information on the entire proton spectral window and the quality of the baseline around the residual water signals in the published studies. As the diagnosis markers for the studied diseases give rise to signals in the aliphatic region of the ^1^H NMR spectrum, most of the published spectra display only the region between 0.0 and 4.0 ppm. Published spectra at 90 MHz, in the 0.0–12.0 spectral window, show a residual water signal covering the region of 3.2–5.2 ppm [69]. At 250 MHz, with a spectral window displaying between 0.0 and 8.0 ppm, the residual water signal masked the region of 4.5–5.5 ppm and the baseline was distorted between 3.5 and 4.5 ppm [57]. At 500 MHz, displayed spectra in the window of 0.0–10.0 ppm showed good baseline and residual water signal masking in the region of 4.3–5.6 ppm in one study [56] and 4.3–5.0 in another study [70]. A study at 600 MHz presented an excellent baseline in the spectral window of 0.0–9.0 ppm, with the residual water signal covering only 4.7–4.9 ppm [58]. Thus, in terms of the quality of the NMR spectra, obviously the main influence is the magnetic field strength. Thus, the reported 90 and 250 MHz spectra are of lower quality than the 400, 500, and 600 MHz ones. As for the 400–600 MHz reported spectra, some differences are seen in their baseline and water suppression quality depending on the generation of the electronics and probe, but all data reported between 1984 and 2023 are well suited for the diagnosis and monitoring of the reported diseases based on targeted biomarkers, with 600 MHz being rather the standard for medical studies with modern spectrometers at the date of the publication of this paper, ensuring a high reproducibility of NMR metabolomics [76,77]. There are also commercial instruments with SOPs and processing solutions available for this latter magnetic field.

**Figure 1:**
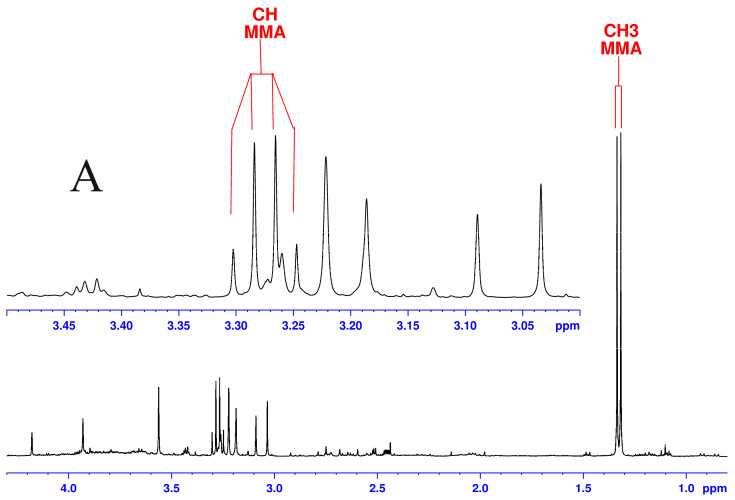

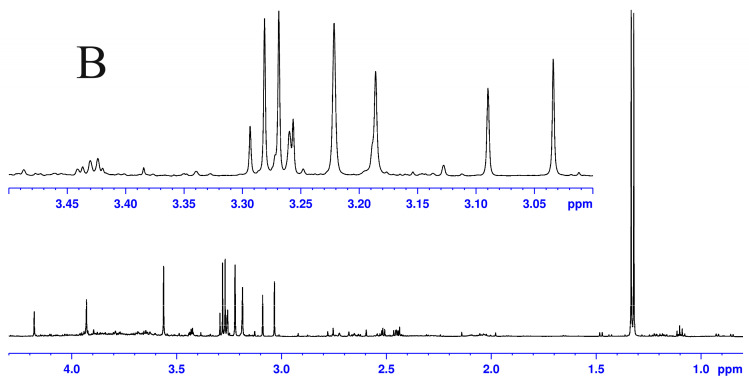
^1^H NMR spectra recorded at 400 MHz (**top**, **A**) and 600 MHz (**bottom**, **B**) from a urine sample belonging to an MMA patient. Both spectra have been recorded with a NOESY presaturation of the water signal, 4 s relaxation delay, 3 s acquisition time, and 32 scans. Deleanu, C. (“C.D. Nenitescu” Inst. of Organic and Supramolecular Chemsitry, Bucharest, Romania) and Nicolescu, A. (“P. Poni” Inst. of Macromolecular Chemistry, Iasi, Romania): Unpublished work.

Figure 1 presents a comparison of one-pulse ^1^H NMR spectra recorded at 400 and 600 MHz from a urine sample belonging to an MMA patient. One can see that methylmalonic acid may be assigned with confidence at both magnetic fields. As the spectra have been recorded from the same sample (same NMR tube), although there is no difference in the chemical shift for the CH group around 3.28 ppm, the apparent widening of the signal at 400 in comparison to 600 MHz is due to the difference in the number of Hz/ppm at different magnetic fields.

Most of the PA, MMA and B_12_ deficiency studies using NMR have been performed on urine samples [33,41,42,43,44,45,46,47,49,50,51,52,53,54,56,57,58,59,69,70,71,72], although a few blood, as DBS [72] and cord blood [74] studies, and CSF [73] studies have been also reported. As expected, the most frequently reported reference for both chemical shift scale and quantitation is sodium 3-trimethylsilylpropionate-d_4_ (TSP) [33,42,44,45,47,49,50,51,52,53,54,55,56,57,58,69,71,73,74]; a few papers have reported sodium trimethylsilylpropanesulfonate-d_6_ (DSS) [43,70] and other compounds such as formate, acetone, lactate and glycine [45], some papers did not report a reference compound [41,46], and one paper reported the electronic referencing signal (ERETIC) as its quantitation reference [58].

Early urine studies relied on HSE [41,42,43,44,45,46,47] spectra, as these spectra support the assignments of signals, but the current method used to record spectra is one-pulse [33,44,45,46,49,50,51,52,53,54,55,56,57,58,59,69,70,71,72,73]. Signals’ assignments have been assisted by COSY [54,55,56,57,58,73] and JRES [48,57] spectra. In some special studies of targeted compounds, additional 2D correlations (HMQC/HSQC, HMBC, TOCSY) have been used [54,55,72]. In blood plasma/serum, CPMG spectra have been used [74].

**Figure 2:**
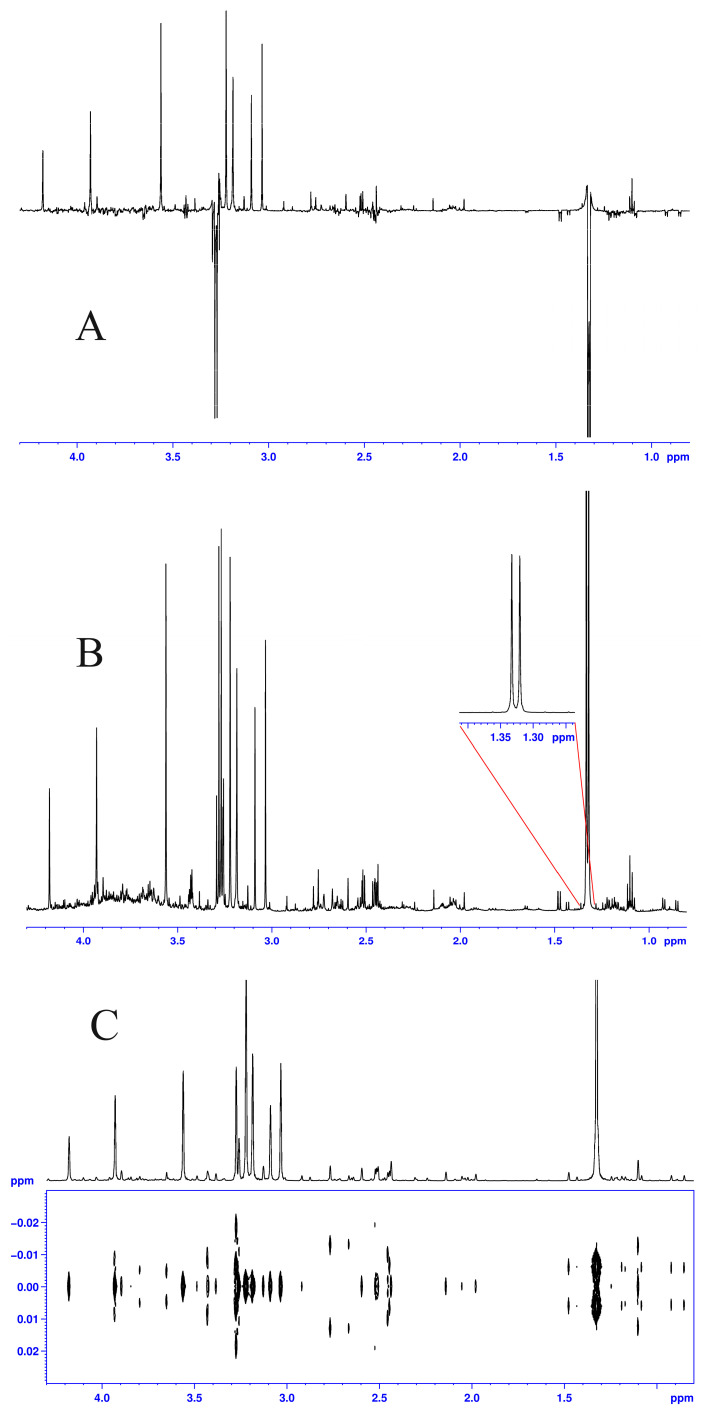
HSE (**top**, **A**), one-pulse ^1^H NMR (**middle**, **B**) and JRES with internal projection (**bottom**, **C**) spectra recorded at 600 MHz from a urine sample belonging to an MMA patient. The 1D spectra in Figure 1A,B have been recorded using comparable parameters, i.e., a 4 s relaxation delay, 3 s acquisition time, and 32 scans. The spectrum in (**A**) was recorded with a HSE pulse sequence employing 60 ms interpulse delays, while the spectrum in (**B**) was recorded with a NOESY presaturation of the water signal. Deleanu, C. (“C.D. Nenitescu” Inst. of Organic and Supramolecular Chemsitry, Bucharest, Romania) and Nicolescu, A. (“P. Poni” Inst. of Macromolecular Chemistry, Iasi, Romania): Unpublished work.

Most of the early assignments of the signals in the ^1^H NMR spectra of urine belonging to PA, MMA and B_12_ patients have been achieved using Hahn spin echo experiments. HSE spectra were widely used in the early days of the ^1^H NMR spectroscopy of body fluids as they help in assigning metabolites due to the fact that signals with an odd number of lines (singlets, triplets, multiplets) are on one side of the spectrum and signals with even numbers of lines (dublets, quartets, multiplets) are on the other side of the spectrum. This was mainly used for assignments, as it was recognized that the integration/intensity of these peaks was no longer proportional to their concentration, preventing direct their quantitation, with the sensitivity (signal-to-noise ratio) being also lower [34,42,47]. Thus, currently, one-pulse ^1^H NMR spectra are almost exclusively used in fast urine analyses. For signal assignments, fast 2D J-Resolved spectra (JRES) are more efficient than HSE. JRES spectra provide both J coupling information on one dimension and chemical shift information for signals with projections of a pure shift type, i.e., resembling a fully decoupled ^1^H NMR spectrum, in the other dimension. Figure 2 exemplifies these types of spectra.

Supplementary Table S1 summarizes the above-mentioned NMR studies.

## 4. Localized In Vivo Nuclear Magnetic Resonance Spectroscopy (MRS)

MRI is able to detect cerebral abnormalities resulting from PA and MMA diseases, but its images are not very specific. Thus, several neurogenetic and metabolic disorders present non-specific brain atrophy in MRI. Most MRI studies have focused on the globus pallidus in the basal ganglia, as they are metabolically very active and are influenced by metabolic abnormalities and toxic poisoning [78]. Thus, some reports determined the basal ganglia to be affected more frequently in MMA than in B_12_ deficiencies [79,80], but other reports seem to contradict this supposed specificity [81,82,83]. Also, it seems that MRI detects abnormalities at a later stage of the disease. Thus, it was reported that 18 children with PA and MMA showed only very subtle abnormalities during their first months of life but developed cerebral atrophy in the next year [84].

MRS could be a more specific and earlier diagnostic tool for these diseases [38,85]. ^1^H MRS can easily monitor N-acetylaspartate (NAA), which is related to neurons’ activity and whose concentration constantly increases from birth until about the age of 20. The other metabolite signals usually detected in MRS are myoinositol (mI), which is thought to protect the brain through its osmolytic property; glutamate, usually overlapped with glutamine (Glx); creatine, usually overlapped with phosphocreatine (Cr + PCr); and choline, usually overlapped with phosphocholine, glycerophosphorylcholine and other choline derivatives (Cho) [50]. ^31^P MRS shows different signals for Cr/PCr and Cho/PCho but no experiments have been reported on our three studied pathologies, thus all MRS references below include only ^1^H MRS. Sherry et al. [39] reviewed brain MRS’s applications in several inborn and acquired metabolic diseases which affect brain metabolism, including MMA and PA [39].

Bergman et al. [86] investigated three PA patients and eight controls using MRS in addition to MRI. MRI showed, in all PA patients, delayed myelination and some cerebral atrophy. In one PA patient with choreoathetosis, MRI exhibited bilateral abnormalities in the image intensity of the putamen and caudate nuclei, while the others exhibited normal basal ganglia. ^1^H MRS of the basal ganglia showed, in all three patients, decreased NAA and mI and an increased glutamine/glutamate ratio [86]. MRS spectra have been measured several times, up to a 26-month interval. A comparison of MRS spectra from a PA patient and a control are presented with their assignments of NAA, creatine (Cr + PCr), choline (“choline-containing compounds”), mI and Glx (glutamine, glutamate and GABA) [86]. The study showed that the MRS information from the tissue (intracellular) could be different to that provided extracellularly by urine, plasma and CSF, which have been analyzed using classical methods, thus underscoring that tissue and body fluids provide complementary information [86].

Lam et al. have compared the MRI and MRS of the basal ganglia of one B_12_-deficient and two MMA patients, along with 17 controls and 10 patients with other types of metabolic disorders [81]. Their levels of mI, choline, creatine and N-acetylaspartate were determined together with the water content of the measured region. N-Acetylaspartate was lower in the B_12_-deficient and one of the two MMA patients. MRS spectra from normal, B_12_-deficient and patients with other pathologies, along with their metabolite assignments, have been presented [81].

Seven controls and four PA patients under treatment (with protein restriction and carnitine supplementation) have been studied by MRI and MRS. The studies were performed from 3 to 5 years after the first diagnosis, when no metabolic decompensation and no or minimal MRI abnormalities were present. MRS revealed lactate in all four PA patients, while none of the controls had lactate. Two of the PA patients who had been diagnosed later also exhibited decreased NAA/Cho ratios, while their NAA/Cr and Cr/Cho ratios were similar to both the controls and PA patients. The MRS spectrum for one PA patient with only elevated Lac, as well as the MRS spectrum for a PA patient with both elevated Lac and a reduced NAA/Cr ratio, are presented [87].

Two MMA cases have been studied by both MRI and MRS [88]. In one patient, in their globi pallidi region, increased Lac and decreased NAA were observed, while their Cr and Cho were reported as normal. In the other regions of the brain with no lesions, concentrations were reported as normal for NAA, Cr and Cho. In the second patient, normal levels of NAA, Cr and Cho, and no detectable Lac, were observed in all regions. However, their Lac was high in all CSF spaces, especially in the lateral ventricles. The MRS spectrum is presented for one patient together with metabolic chemical shift images for their NAA and lactate (NAA-CSI and Lac-CSI MRI). For the second patient, their Lac-CSI is also presented [88]. The study highlighted the superiority of the newer multi-slice MRS and diffusion-weighted MRI over previous techniques, as multi-slices may cover regions unsuspected by MRI for abnormalities [88]. This study confirmed earlier observations that the Lac concentration is not correlated between the brain tissue (intracellular) and CSF (extracellular) [86].

The monitoring of an MMA patient before and during therapy with carnitine and vitamin B_12_ was followed by MRI and MRS [89]. MRI showed high signals in both their basal ganglia. MRS revealed high lactate and decreased NAA in their MRI regions, with high intensity. The MRI images and MRS spectrum of basal ganglia are presented [89]. MRS images recorded before the initiation of the treatment and periodically up to 9 months during therapy revealed a constant decrease in lactate until its complete disappearance and constant increase in their NAA level [89].

A nutritional B_12_ deficiency in a breast-fed infant due to maternal malabsorption was studied by both MRI and MRS [90]. MRS showed, during the first examination, high lactate in the supratenorial gray and white matter. Low choline and inositol and normal levels of NAA and other metabolites were observed in the white matter. Lower NAA, Cr, Cho, mI, Glu and Gln and higher Lac were observed in the gray matter. During the second examination, six months later, after B_12_ therapy, although MRI was still showing an intense signal in basal ganglia, in MRS all metabolites in both the gray and white matter returned to their normal values and lactate was no longer present. The normalization of the MRS spectrum matched the return to the normal level of methylmalonic acid in the urine and a general improvement in all clinical, including mental, aspects. This showed the reversibility of the condition when nutritional B_12_ deficiency is treated in its early stages [90].

In an MMA with homocystinuria (MMA-HC) study, five patients were monitored by both MRI and MRS [83]. Three patients showed, in MRI, lesions in their basal ganglia and one showed a subependymal hemorrhage. MRS performed during the chronic phase of the disease revealed high lactate in the basal ganglia in two cases, while, in one case, lactate was present in the periventricular white matter corresponding to the MRI lesions. In spite of the fact that all patients had high concentrations of methylmalonic acid in their urine, their MRS spectra were normal in both cases, with normal MRI. The ratios of choline/creatine, NAA/creatine and NAA/choline were normal in all but one patient, who showed increased choline/creatine and decreased NAA/choline [83].

Valayannopoulos et al. [91] studied, by MRS and MRI, three patients with succinyl-coenzyme A synthetase deficiency (SCS) combined with encephalomyopathy and mild MMA. The paper revealed that SUCLG1 mutation is associated with the excretion of methylmalonic acid and C4-DC carnitine in urine and lesions of the basal ganglia visible in MRI. All three patients showed T2 and FLAIR-MRI-enhanced intensities in their bilateral caudate nuclei and bilateral putamen. Lactate was present in the basal ganglia of two patients [91].

Davison et al. [92] presented an MRS study of eight children with PA, with experiments performed both during metabolic stability and acute encephalopathic episodes. Two children who had previously had liver transplants did not show significant differences from the control group, supporting, thus, the protective benefit of transplants. MRS quantitative data have been compared with a large control cohort, making this study a valuable reference for PA MRS. The paper describes significant decreases in the basal ganglia of Glx (combined glutamine + glutamate) and N-acetylaspartate, together with increased lactate during encephalopathic episodes, whereas, in the white matter, only lactate was increased. During decompensations, separate glutamine and N-acetylaspartylglutamate (tNAA) levels were also decreased in the basal ganglia. Published, averaged spectra for metabolically stable PA versus acute encephalopathy episodes clearly underlines the power of MRS in differentiating these states. MRI during severe episodes showed abnormal signals in the basal ganglia, while MRI was normal during metabolic stability [92]. It was also suggested that the better quantitation method used in this study [92] would contradict a previous study where Glx was estimated to increase in PA [86].

Nine patients with combined MMA and homocystinuria (MMA-HC) have been examined by MRI and MRS, and they were under a specific treatment during examination [93]. The diagnosis of MMA-HC was confirmed by genetic tests. All patients were diagnosed by inborn screening and started treatment as early onsets. Patients were also subjected to extensive neurological and neurodevelopmental evaluation protocols (either in their native language or with an interpreter). The most frequent abnormalities have been listed as a craniocaudally short pons, callosal thinning and enhanced T2 FLAIR signal in the periatrial and periventricular white matter. Basal ganglia MRI spectra were assessed as normal, and MRS spectra were also assessed as normal, in all patients, despite specific markers (methylmalonic acid, methionine, homocysteine and propionylcarnitine) in their urine and plasma. Thus, guanidinoacetate was not seen and creatine was not decreased in MRS. The authors confirmed the previous assumption that plasma concentrations of MMA and homocysteine in early infancy are not well correlated with neurodevelopmental outcomes. No MRI images, no MRS spectra, and no information on the equipment used were reported [93].

Engelke et al. published in their book [36] on the brain MRS spectrum of an MMA patient with methylmalonic acid identified in the spectrum. This is one of the rare spectra where the authors, apart from lactate, identified, using MRS, the methylmalonic acid signal [36].

Ekici et al. [94] investigated by MRI and MRS 14 patients with nutritional vitamin B_12_ deficiency. Four cases were followed up for several months. Eight cases showed abnormal MRI, with most common issues being a thinning of the corpus callosum and brain atrophy. In MRS, lactate was not present in any patients and the ratios of NAA/Cr and Cho/Cr were assessed as normal after treatment. The values of the brain NAA/Cr and Cho/Cr ratios, together with the vitamin B_12_ concentrations in their blood, are reported. Pre- and post-treatment MRI and MRS images and spectra are presented [94].

Kayal et al. [95] performed a study aiming to establish the cause of several non-compressive myelopathies in a tertiary care hospital in India. Thus, 55 patients with non-compressive myelopathies were diagnosed, after the MRI of their spine, with chronic myelopathy (CM). In four of these patients, the cause of their CM has been identified as a vitamin B_12_ deficiency. Spinal cord MRI was performed on all patients and brain MRI was performed on selected patients. The level of vitamin B_12_ in their serum was also analyzed in all patients. All B_12_ patients had a good outcome after metabolic supplementation. The causes of vitamin B_12_ deficiency were determined to be combinations of a strict vegetarian diet, malnutrition, alcohol consumption, and phenytoin therapy. Although MRS was mentioned as a performed analysis, no details have been given and only MRI details and images have been presented [95].

Twenty-eight combined methylmalonic and homocystinuria (MMA-HC) patients and twenty-one controls were examined via both MRI and MRS [96]. All patients were examined by MRI and MRS before starting treatment. The patients showed different MRI patterns of abnormalities, with main features being hydrocephalus and supratentorial white matter edemas. None of the patients exhibited abnormal MRI signals in their basal ganglia, and this was mentioned by the authors as a distinctive feature of MMA-HC in comparison with isolated MMA and PA. Three patients with no lesions in their basal ganglia, as shown by MRI, exhibited a Lac signal in the MRS of these regions. The published MRI data exemplify two cases with normal signals in their bilateral basal ganglia and MRS spectra showing a lactate signal in their bilateral basal ganglia. The ratios of NAA/Cr and NAA/Cho were decreased in all patients and lactate was present in three patients. No abnormal values were noticed for the ratios of Cho/Cr and mI/Cr. Moreover, it was shown that there were no Cho increases in patients, thus the reductions in the NAA/Cho ratio were solely generated by the decrease in NAA. There were no correlations between NAA/Cr values and any biochemical markers in their blood [96].

Eighteen B_12_-deficient patients without developmental delays and twelve controls were examined by MRI and MRS in three different regions of their brain in order to evaluate the levels of their brain metabolites. All MRI and MRS evaluations were performed before starting treatment. MRS spectra were recorded from three different brain parenchyma regions (i.e., the left frontal subcortical, left lentiform nucleus and left cerebellar dentate nucleus), as they have been known to be affected by vitamin B_12_ deficiencies. MRI revealed periventricular white matter hyperintensities in two patients and brain atrophy in four patients. Lactate was not present in any of the subjects. The ratios of NAA/Cr, Cho/Cr, mI/Cr and Glx/Cr were evaluated for all participants and the authors reported that there were no differences in any of the metabolite ratios between B_12_-deficient patients and the controls. The concentration ratios for these metabolites in all three studied regions, for both the pathological and control groups, are presented [97]. The authors indicated that their result is similar to a nutritional study in which healthy voluntaries were administrated high doses of B vitamins and no metabolic differences were observed between the supplemented and placebo groups [98]. In spite of this conclusion, the authors reported that, for the only two patients who were examined by MRS before and after starting treatment, their ratios of NAA/Cr, Cho/Cr and mI/Cr were increased, while that of Glx/Cr decreased [97]. We may thus add to their conclusion that although the concentration ranges were similar, a positive trend could be assessed when looking retrospectively at the same subject. Obviously, one cannot draw conclusions from only two cases. High-quality MRI images are included in the paper, but no MRS spectra are presented [97].

A cohort of 35 patients with a vitamin B_12_ deficiency have been followed up by MRI and MRS (only 24 by MRS). There was no control group in the study. Twenty-six patients had abnormal MRI. Most of the patients’ mothers were reported to be vegetarian. All patients had neurologic symptoms as well as low serum vitamin B_12_ concentrations [99]. Brain images were presented but no MRS spectra were shown. MRS was recorded in the left periventricular frontal white matter region and basal ganglia (left lentiform nucleus). The ratios of Cho/Cr and NAA/Cr have been measured by MRS and their values are reported. Graphic correlations of MRS-determined metabolites’ ratios with motor/mental development, as well as their correlations with blood levels of vitamin B_12_ and homocysteine, are presented. No correlation between these parameters has been found, although, in 74% of cases, neuroimaging abnormalities were identified [99].

## 5. ^1^H MRS Methodology

The studies reviewed above have recorded MRI and MRS spectra at magnetic fields of 1.5 Tesla (64 MHz proton frequency) [36,80,81,83,85,86,87,89,91,92,95,96,97,99]. In a few studies, the magnetic field was not mentioned [82,88,90,93], but we may assume that the field was also 1.5 Tesla. Only one study was performed at 3 Tesla (127.7 MHz proton frequency) [94].

In terms of the selection of the region for recording the MRS spectra, most of the studies used single-voxel spectroscopy (SVS) with point-resolved spectroscopy (PRESS) or a stimulated echo acquisition mode sequence (STEAM) in the regions selected as abnormal by the MRI images [36,81,86,87,89,90,92,94,96,97,99].

In a few cases, the selection technique was not mentioned, but we may assume that it was also SVS as, for instance, one study mentioned MRS of the basal ganglia (we assume single-voxel) [80,82,91,93,95].

In one case, MRI and MRS data have been reported and commented on, but no MRI images, no MRS spectra and no information on the equipment used were reported [93].

Modern multi-slice recording with chemical shift imaging (CSI) was used in some of the reported studies [88], and this allowed for clear metabolic images based on choline (Cho), creatine (Cr), NAA and lactate to be reconstructed. Surprisingly, as mentioned above, although the authors gave details on their experimental conditions, no mention of the instrument type and magnetic field was given in this paper [88]. One paper, which involved studies over a longer period of time, mentioned experiments performed with two techniques as they most likely changed their instrument [87]. Thus, MRS experiments before 1996 were performed using SVS with STEAM, and those after 1996 with a CSI pulse sequence. Also, as no absolute quantification was available until 1996, the authors have presented their results as metabolite ratios of NAA/Cho, NAA/Cr and Cr/Cho [87]. Another paper also mentioned both techniques, i.e., CSI for some patients and SVS for others [83].

Similarly to the situation in the previously discussed NMR studies, for MRS, the published spectra display only the region of 0.0–4.5 ppm, which is relevant for markers of the discussed pathologies, skipping, thus, the residual water signal and the aromatic region.

**Figure 3:**
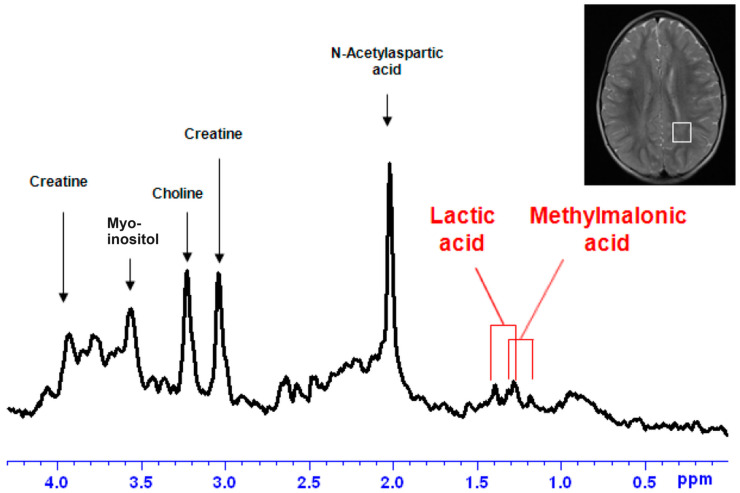
MRS spectrum recorded from the selected MRI voxel at 1.5 Tesla from a patient with MMA due to a methylmalonyl-CoA mutase deficiency. Reproduced with permission from Prof. Ron A. Wevers [36].

Figure 3 exemplifies an MRS spectrum recorded using PRESS at 1.5 Tesla from an MMA patient. This is one of the rare published spectra in which methylmalonic acid is identified in an MRS spectrum.

Supplementary Table S2 summarizes the above-mentioned MRS studies.

## 6. Conclusions

Both ex vivo, non-localized NMR and in vivo, localized MRS have been proven to be valuable tools in exploring MMA, PA and B_12_ deficiencies, as well as IEM in general. While the NMR of body fluids provides better resolution, signal-to-noise ratios and more reliable quantitation, the MRS of tissue provides complementary information. Thus, it was recognized that there are different levels of the same metabolite intracellularly in the brain tissue and extracellularly in the body fluids (CSF, plasma, urine), and these levels are not always correlated with the severity of the disease or treatment efficacity. However, the joint information provided by both NMR and MRS in the same patient is very much underexplored. Particularly, the joint use of MRS of the brain and NMR of the CSF have a great potential to advance diagnosis and knowledge in general, but the quite invasive nature of CSF sampling has prevented this development. However, considering both the life-threatening complications in severe cases of IEM and the need for more efficient treatments, the use of these combined techniques could be a good choice in the future.

One study using ^1^H NMR on cord blood revealed markers for the potential risk of MMA in gestational hypothyroidism (GHT). Although the prenatal diagnosis of these conditions via a classical analysis of amniotic fluid is possible, no attempt to explore the potential of NMR in this respect has been published.

At least for the disorders covered by this review, routine NMR spectrometers with magnetic fields equal to or higher than 400 MHz, and without special accessories, can easily diagnose these diseases and monitor their therapies.

Once one of these diseases is already diagnosed, NMR spectroscopy may easily and efficiently monitor its therapy on long term, and this is possible even when a laboratory is not located in the vicinity of the clinical site. Thus, for routinely monitoring these diseases, NMR is the choice, as MRS requires the patient instead of the sample and it involves much higher costs per analysis. For both NMR and MRS, longer-term monitoring requires an adjustment of the results with control groups of a corresponding age, as in neonates and young children normal metabolic ranges are shifting very fast.

^31^P MRS was not used for these diseases, although metabolites as phosphocreatine, inorganic phosphate and ATP could be easily detected and might be altered in these pathological states.

In spite of the growing popularity of functional MRI (fMRI), there are no reported studies using fMRI to determine the neurocognitive particularities of any of the three reviewed diseases.

Current 3-Tesla or higher magnetic field instruments should enable better signal separation (e.g., glutamine versus glutamate) and possibly more specific patterns for the reviewed pathologies. With 1.5 Tesla instruments, most metabolic alterations have been followed for only a small number of metabolites which have common patterns for several pathologies. This limitation at our current stage of knowledge makes it a necessity to interpret MRS information in complement with other physiological and biochemical data. However, the highest clinical commercially available magnetic field has already reached 7 Tesla, and research on whole-body human systems already exists using 11.7 Tesla. These high-field systems could be a game changer in the MRS of IEM.

An extensive MRS comparison of non-transplanted versus transplanted patients could also help in clarifying the current controversy related to the benefits of liver transplantation in PA patients.

With the existing published data, both the MRI and MRS of these pathologies reflect rather non-specific changes, making, thus, clinical laboratory investigations or NMR the primary diagnosis tools. As the primary diagnosis of these pathologies is based on plasma and urine markers, followed by enzyme activity in cultures, the potential of NMR diagnosis, which is highly specific, is much underused, at least in suspicious cases. Similarly, MRS is much underused, although modern MRI instruments can easily add this information to the same patient investigation.

In terms of interpreting raw data, although less popular to date, NMR and MRS are straightforward in providing either absolute or relative metabolite concentrations, whereas interpreting MRI, although widely accepted in medical diagnoses, has an important subjective component. Thus, in several of the above-mentioned studies, for MRI interpretations, two neuroradiologists have independently analyzed the MRI data.

In conclusion, fMRI, higher magnetic field MRS with multi-slice CSI, and a combination of intracellular MRS information with extracellular NMR (particularly of CSF) are all largely un- or underexplored in connection with MMA, PA and vitamin B_12_ deficiencies, and it is very likely that these techniques or combinations of techniques will provide new insights into the mechanisms of these diseases, as well as new diagnosis tools. In terms of neonatal screening, NMR has proven its potential, but its use in combination with MS is also scant. The discussed pathologies are notably extremely rare, and with the mentioned advances in technology there is room for future leaps in our knowledge of these pathologies.

## Supplementary Materials

The following supporting information can be downloaded at https://www.mdpi.com/article/10.3390/biom14050528/s1, Table S1: Summary of non-localized NMR studies on MMA, PA and B_12_ deficiencies; Table S2: Summary of localized MRS and MRI studies on MMA, PA and B_12_ deficiencies.
