# Supplementary material for: NMR Spectroscopy in Diagnosis and Monitoring of Methylmalonic and Propionic Acidemias
Source: Biomolecules. 2024 Apr 28;14(5):528. doi: 10.3390/biom14050528 (PMC11117674; doi:10.3390/biom14050528)
Supplement: Supplementary file 1 [file biomolecules-14-00528-s001.zip › Supplementary-Table-S1.pdf]

NMR spectroscopy in diagnosis and monitoring of methylmalonic and propionic acidemias

Calin Deleanu and Alina Nicolescu *

Supplementary 1

Table S1. Summary of non-localized NMR studies on MMA, PA and B_{12} deficiency.

Key aspects	Sample preparation	NMR Experimental details	Disease	Mentioned metabolites / Features	Cohorts	Ref.
First reports on NMR detected MMA and PA. Stated that metabolites at conc >0.5 mM visible in NMR in 5 min. For diagnosis of studied acidurias spectra may be used as fingerprints.	Urine + 10% D_2O with DSS.	400 MHz (Bruker WH400).<br>HSE (interpulse delay τ=60 ms, 1 s recycling delay, water supp., 240-360 scans, total time 4-6 min).<br><br>HSE of normal and another IEM presented.	MMA, PA, other IEMs, control.	Peaks identified in normal urine included creatinine, citrate, hippuric acid and various amino acids.	N/A Proof of concept study.	[41], [42]
First reports on NMR detected MMA and PA.	Urine + 10% D_2O	400 MHz (Bruker WH400).<br>HSE (τ=60 ms, 1 s recycling delay, water supp.),<br>One pulse (2 s recycling delay)<br><br>No spectra shown.	MMA, PA, other IEM	MMA: Methylmalonate with variable glycine, 3-hydroxypropionate or propionylglycine. Progress of a ketotic episode lasting 5 days followed by changes in acetone, 3-hydroxybutyrate and 3-hydroxypropionate.<br>PA: Large fluctuations of glycine, propionylglycine, 3-hydroxypropionate, and acetate.	One MMA monitored 12 days.<br>One PA monitored over several month.	[46]
First NMR spectra presented with assignments for MMA and PA.	Urine + 10% D_2O with DSS.	400 MHz (Bruker WH400).<br>HSE (τ=60 ms, 1 s recycling delay, water supp. 200 scans for PA, 64 scans for MMA).<br><br>HSE spectra with assignments presented for one PA and one MMA.	MMA, PA	In PA conjugate of propionyl-CoA and propionylglycine assigned and noticed in high amounts. Glycine mentioned “in large amounts” and 3-hydroxypropionate as “clearly visible”. A variable NMR fingerprint for PA urine was mentioned. Typically, 3-hydroxypropionate and/or propionylglycine have been visible although sometimes none of them was detectable. High glycine in all PA. In a	5 MMA, several PA (one monitored over 10 months).	[43]

Key aspects	Sample preparation	NMR Experimental details	Disease	Mentioned metabolites / Features	Cohorts	Ref.
				MMA with ketoacidosis high amounts of methylmalonic acid, 3-hydroxybutyrate, and creatine. In the other MMA patients methylmalonic acid was always present with only occasional ketosis episodes. Spectral assignments for: acetone; acetate; creatine; creatinine; glycine; lactate; methylmalonate; 3-hydroxybutyrate; 3-hydroxypropionate; propionylglycine.		
First mention of One pulse for quantitation and HSE for assignments; Detailed chemical shift signal assignments; Estimated that metabolites present in concentrations higher than 0.5 mM can be detected at 400 MHz and that higher field spectrometers will improve diagnosis in metabolic diseases. First time good correlation between NMR and GC-MS for the metabo-lite/creatinine (Crn) ratios was also documented.	Urine + 10% D_2O. TSP and formate as both chemical shift and quantitation reference or added acetone as chemical shift reference, as well as endogenous lactate or glycine also used as chemical shift reference. Detailed techniques, including spiking, as well as lyophilization and redisolvation in D_2O have been described.	400 MHz (Bruker WH400).<br><br>HSE (τ=60 ms, 1 s recycling delay, 0.95 s acq. time, water supp.).<br>One pulse (5 s recycle delay, 60 to 400 scans).<br>Recorded at 20°C.<br><br>Good quality HSE spectra for controls, and One pulse for PA and MMA presented.	MMA, PA, IEMs, normal	In MMA: high concentrations of 3-hydroxybutyrate, acetone and isovalerylglycine reported with markers well assigned in NMR spectra. In PA: assignments for high concentration specific markers reported for 3-hydroxypropionate, propionylglycine, propionylcarnitine, glycine variable propionylglycine or 3-hydroxypropionate and tiglylglycine. Methylcitrate first time reported by NMR in PA but only visible in lyophilized extracts.	4 PA, 5 MMA, 7 IEMs, several controls.	[45]
Correlations between NMR and GC-MS for the metabolite/creatinine ratios. First detailed NMR/MS comparison for MMA and PA. First example of detailed NMR metabolomic monitoring of PA and MMA during treatment.	Urine + 10% D_2O. Either sodium formate or TSP-d_4 as a chemical shift reference and standard for quantification.<br>To some samples from the patients with PA, acetone	400 MHz (Bruker WH400).<br><br>HSE (60 ms interpulses delay, 80-400 scans, recycle delay 2 s).<br>One pulse (45°, 80-400 scans, recycle delay 2 s or 5 s for quantitation).<br>Recorded at Room temp.	MMA and PA patients during metabolic decompensat ions.	Metabolites assigned: methylmalonic acid, 3-hydroxypropionate, propionylglycine, propionylcarnitine, glycine, propionylglycine, 3-hydroxypropionate, 3-hydroxybutirate, tiglylglycine, methylcitrate, cratine, betaine, creatinie, acetone, acetate, hippurate.<br>Concentrations relative to creatinine are given for betaine, creatine, glycine, 3-	Monitoring a PA patient over 10 months and a MMA patient over 11 days. For comparison other 4 PA and 4 MMA patients with only one time analysis	[44]

Key aspects	Sample preparation	NMR Experimental details	Disease	Mentioned metabolites / Features	Cohorts	Ref.
	was added as a secondary chemical shift reference.	Signal assignments also assisted by ion-exchange chromatography separations.<br><br>HSE and one pulse spectra with assignments for MMA and PA are shown.		hydroxypropionate, propionylglycine, methylmalonic acid, 3-hydroxybutyrate.	have been included.	
Report stating that HSE fingerprint for MMA should be definitive but the PA fingerprint is quite variable even for the same patient. Recognized that integration/intensity of peaks in HSE is not proportional to concentration, preventing direct quantitation and also with sensitivity (signal-to-noise ratio) being lower.	Urine + 10% D_2O + TSP-d_4.	400 MHz.<br>HSE (60 ms interpulse delay, water supp.).<br>One pulse (30° pulse, 2s recycling delay, water supp.).<br><br>HSE spectra for MMA and PA presented with assignments.	MMA, PA	Carnitine/acylcarnitine, creatinine, creatine, glycine, methylmalonate, 3-hydroxypropionate, propionylglycine, tiglyglycine+tiglate have been assigned in the HSE.<br>HSE fingerprint for MMA should be definitive but the PA fingerprint is quite variable even for the same patient.	One MMA, one PA.	[47]
Normal fasting and diabetes.	Urine + 10% D_2O + TSP-d_4	400 MHz.<br>One pulse (30° pulse, 5s recycling delay, 32 scans, water supp.)<br>One pulse spectra for a fasting control with assignments presented.	Normal, another metabolic disorder.	Acetyl carnitine is present together with ketone bodies in 48 hours fasting control but not present after 12 hours fasting.	12 and 48 hours fasting control.	[33]
The 90 MHz published spectra with limited diagnosis value. Nevertheless, the paper shown that even with such low field a MMA suspicion can be quickly raised by NMR.	Urine + 10% D_2O + TSP-d_4.	90 MHz (2.1 Tesla) (Jeol FX900).<br>One pulse, 45°, recycling delay 8 s, water supp., 150 scans, total recording time 20 min).<br><br>One pulse spectra presented for MMA, control, another IEM.	MMA, another IEM, controls.	Only the methyl doublet of the methylmalonic acid is a distinguishable feature in the published spectrum.	One MMA in comparison with another IEM pathology, and 35 controls.	[69]
Diagnosing various metabolic disorders. Admitted that 250 MHz could be a too low frequency for most of the studies.	Urine + 10% D_2O + TSP-d_4, adjusted to pH 2.5 with HCl.	250 MHz (5.9 Tesla) (Bruker).<br>One-pulse (90° pulse width, presaturation during 6 s relaxation delay, 80 scans, 12 min recording time).<br>Mentioned JRES and COSY.<br>Diagnoses confirmed by GC-MS.	MMA, PA, control and other IEMs	A list of chemical shifts for relevant metabolites (including methylmalonic acid and glycine, 3-Hydroxypropionic acid, tiglylglycine)	3 MMA, 2 PA, other IEMs, one control.	[57]

Key aspects	Sample preparation	NMR Experimental details	Disease	Mentioned metabolites / Features	Cohorts	Ref.
Detailed monitoring of several metabolites in PA and MMA patients under L-carnitine therapy.	Urine + 10% D_2O + TSP-d_4.	250 MHz and 400 MHz (Bruker AM250 and WH400).<br>One-pulse (45° pulse angle, 2s recycle delay, water presaturation during relaxation delay; 128, 168, 237 256, 288 and 414 scans).<br><br>250 MHz mentioned but not shown.<br><br>Well resolved MMA and PA assigned one-pulse NMR spectra recorded at 400 MHz have been presented.	MMA, PA, Control.	In PA: betaine, creatine, carnitine, propionylcarnitine and "total propionate" (i.e. 3-hydroxypropionate + propionylglycine + tiglylglcyine + propionylcarnitine) over up to 22 hours after L-carnitine administration have been presented as concentrations relative to creatinine. In MMA: propionylcarnitine, acetylcarnitine, and "total propionate" (i.e. methylmalonate + propionylcarnitine + 3-hydroxypropionate) over 30 hours after L-carnitine administration have been presented as concentrations relative to creatinine.	Two PA and one MMA patients under L-carnitine therapy monitoring.	[49]
Control and MMA monitored after carnitine intravenous.<br>Mentioned as unpublished result the detection by ^1H NMR of 2,3 butandiol in the urine of some PA patients but no details or spectra have been published.		400 MHz and 500 MHz.<br><br>One pulse spectra with assignments.	PA, MMA, Control, other IEMs	Assigned: acetylcarnitine, carnitine, creatine, creatinine, glycine, methylmalonate, 3-hydroxybutyrate, 3-hydroxypropionate, propionylcarnitine, acetate, alanine, betaine, citrate, dimethylamine, dimethylglycine, lactate.	One control; One MMA monitored after carnitine intravenous.<br>Other IEMs.	[50] Review
Monitored two MMA patients and two other IEMs during metabolic decompensation episodes.	Urine + 10% D_2O + TSP-d_4.	250 MHz and 400 MHz (Bruker AM250 and WH400).<br>One pulse (45° pulse angle, 2 s recycle delay, water presat. during relaxation delay) at 20°C.<br><br>No spectra have been presented.	MMA	Increased both creatine/creatinine ratio and total daily creatine during decompensation. Return to normal during recovery. Absolute methylmalonic acid conc not fluctuating during decompensation although the methylmalonic acid/creatinine was fluctuating. Evolutions of creatine, 3-hydroxypropionate, 3-hydroxybutyrate and methylmalonic acid presented as graphics relative to creatinine. Also presented, graphics relative to creatinine for creatine, 3-hydroxypropionate, propionylcamitine, acetoacetate, acetone, 3-hydroxybutyrate and methylmalonic acid during carnitine treatment.	2 MMA, 2 other IEMs during metabolic decompensation episodes.	[51]
Differentiating diagnosis of MMA from PA. Higher	Urine + 10% D_2O + TSP-d_4.	500 MHz (11.7 Tesla) (Varian VXR500).	MMA, PA	Quantified methylcitrate in native urine of a PA, in addition to 3-hydroxy-n-valerate,	One MMA, one PA	[56]

Key aspects	Sample preparation	NMR Experimental details	Disease	Mentioned metabolites / Features	Cohorts	Ref.
magnetic field and additional COSY experiments have been employed to directly quantify methylcitrate in native urine of a PA patient. Ratios of metabolites to creatinine reported to be in good agreement with GC-MS results.		One-pulse (90° pulse, 32-160 scans, water presat. during 6 s relaxation delay), and COSY.<br><br>Well resolved 500 MHz one pulse spectra of urine from PA and MMA patients with assignments of methylmalonate, glycine, creatinine, creatine and betaine have been presented. Also COSY for PA is presented.		propionylglycine, 3-hydroxy-n-butyrate, lactate, tiglylglycine, acetoacetate, 3-hydroxypropionate, betaine, glycine, hippurate, creatinine, and propionyl carnitine. Concentrations relative to creatinine reported before and after L-carnitine treatment. Concentration of methylmalonate reported before and after treatment with L-carnitine and sodium benzoate.		
Monitoring of metronidazole treatment in PA and MMA before and up to 20 days after beginning the metronidazole treatment. The study revealed that although the total propionate was significantly reduced by metronidazole, the propionylcarnitine was not decreased	Urine + 10% D_2O + TSP-d_4, at pH 6.0.	500 MHz (Jeol GSX500).<br>One pulse (30-45° pulse width, 2s water presat., 5s recycling delay).<br><br>Well resolved 500 MHz 1H NMR spectra with assignments of PA before and after administration of metronidazole are presented.	MMA, PA	Total propionate in PA reported as sum of propionylcarnitine, 3-hydroxypropionate, methylcitrates, tiglylglycine and propionylglycine. In MMA methylmalonic acid was added to the previous calculation in order to account for total propionate.	Two PA and two MMA monitored before and up to 20 days after beginning the metronidazole treatment.	[52]
Brief communication. No MMA spectrum was presented. They have also reported neural network classification without further details	Urine + 20% D_2O + DSS	500 MHz ("Varian instrument").<br>One pulse (62 scans, 6 s repetition time, water suppression) at 298K,<br><br>Spectra of other IEMs presented but no MMA spectrum presented.<br><br>Reported neural network classification without further details	MMA, other IEM.	Neural network classification of several controls and IEMs, including MMA, without further details was reported	85 patients with confirmed IEMs, including MMA.	[70]
NMR spectra have been discussed in comparison with desorption electrospray ionization mass spectrometry (DESI-MS). Principal component analysis (PCA) was used for separating spectra and for assisting in identifying	Urine + 10% D_2O + TSP + phosphate buffer with pH 7.1.	500 MHz ("Bruker").<br><br>One pulse (30° pulse, noesy presaturation, 32 scans) at 298 K.<br><br>NMR discussed in comparison with desorption electrospray ionization MS (DESI-MS).	MMA, controls	A list including NMR chemical shifts and MS fragments mass to charge ratios (m/z) for several normal and pathologic metabolites was presented. A correlation matrix chemical shift versus m/z is also presented. no concentrations have been provided although specific markers for MMA have been listed as methylmalonate,	20 controls, one MMA, 5 other types of IEMs.	[53]

Key aspects	Sample preparation	NMR Experimental details	Disease	Mentioned metabolites / Features	Cohorts	Ref.
specific markers based on loading plots. The authors advocate the advantage of performing urine screening by combined NMR and MS techniques. The paper recognized that it is not possible to determine concentrations from PCA outputs but it pointed out that targeted metabolite concentrations may be obtained from the original ^{1}H NMR spectra. Although the paper showed the possibility of PCA to separate MMA form controls and other IEMs, due to the small number of spectra in each group this is not convincing.		A small MMA spectrum without details is presented.		trimethylamine N-oxide (or betaine), and dimethylamine, together with three unidentified singlets. However, no concentrations have been provided although specific markers for MMA have been listed.<br>Principal component analysis (PCA) was used for separating spectra and for assisting in identifying specific markers based on loading plots.		
Unambiguously identifying the two methylcitrate isomers in native urine of PA and MMA patients. A well resolved urine PA one-pulse ^{1}H NMR spectrum with assignments together with the HMQC correlations are presented	Urine + 10% D_2O + TSP-d_4 at pHs of 2.5 and 12.5 adjusted with HCl and NaOH.	400 MHz (Varian Mercury Vx)<br>One pulse spectra (30° pulse width, 3s acquisition time, water suppression during 5s relaxation delay, 1000 scans).<br>Additional COSY, HMQC, HMBC on standard methylcitrate and PA.<br><br>A well resolved one-pulse spectrum with assignments and HMQC for PA are presented.	PA, MMA, standard compounds	SS and RS methylcitrate isomers	N/A	[54], [55]
Several reference ^{1}H NMR spectra of urine form a PA and three types of MMA.			PA, MMA		N/A	[36] Book
It was shown that in addition to therapy assessment, the levels of methylmalonic acid and glycine in urine can indicate	Urine + 10% D_2O + TSP-d_4 with phosphate buffer.	400 and 600 MHz (9.4 and 14.1 Tesla) (Bruker Avance Neo 400 and 600).	MMA	Absolute and relative to creatinine concentrations for a comprehensive set of 26 metabolites (including glycine,	5 MMA patients for several months up to 9 years intervals.	[58]

Key aspects	Sample preparation	NMR Experimental details	Disease	Mentioned metabolites / Features	Cohorts	Ref.
the type of MMA mutation (MMAA *versus* MMUT).	ERETIC signal as quantitation reference in addition to TSP-d_4.	Fast one pulse spectra (32 scans, 90° pulse width, 4 s relaxation, noesy presat, 2.7 s acquisition time).<br>Fully relaxed one pulse spectra (32 scans, 90° pulse width, 30 s relaxation time, 3 s noesy presat, 4 s acquisition time).<br>JRES and COSY used for accurate signal assignments.<br>A 600 MHz one-pulse spectrum with assignments, a JRES and a COSY from a MMA case are presented as examples.		methylmalonic acid, orotic acid, orotidine, L-carnitine and propionylcarnitine).<br>Methylmalonic acid and glycine concentrations in urine have been associated with the type of MMA mutation (MMAA versus MMUT).<br><br>Control ranges from literature data also presented.		
A qualitative differentiation based on combined ^{1}H NMR profile at 400 MHz and GC-MS, between several IEMs, including PA.	Urine + 25% D_2O + TSP-d_4 with phosphate buffer with pH 7.	400 MHz (9.4 Tesla) (Bruker Avance).<br>One pulse (noesy presat, 64 scans, 4 s relaxation delay, 3.41 s acquisition time) at 296 K.<br><br>An NMR spectrum from a control, together with spectra for various IEMs, including one PA spectrum are presented.<br><br>Multivariate statistical analyses have shown some separation from healthy and IEMs, but the number of data was small and results are not very convincing. Multivariate statistical study on ^{1}H NMR and GC-MS data has shown some separation from healthy and IEMs, but the number of data was small and results are not convincing.	PA, controls, IEMs	For PA specific metabolites have detected by NMR: propionic acid, 3-hydroxypropionic acid, and propionylglycine, while by MS the specific metabolites have been identified: 3-hydroxypropionic acid, 3-hydroxyvaleric acid, propionylglycine, tiglylglycine, and methylcitric acid. A list of NMR chemical shifts for various metabolites in controls and specific IEMs are presented, but no concentration information is given.	36 controls, 2 PA, several other IEMs	[71]
Demonstrated that the dry filter paper technique is well suited for NMR diagnosis, at least for	Dry filter paper saturated with urine.	500 MHz (Bruker DRX 500).	MMA, controls, other IEMs.	Good reproducibility of concentrations relative to creatinine for alanine, threonine, citrate, hippuric acid, methylmalonic acid, propionyl carnitine, as well as 6 other	5 controls, 1 MMA, 3 other IEMs.	[59]

Key aspects	Sample preparation	NMR Experimental details	Disease	Mentioned metabolites / Features	Cohorts	Ref.
controls and studied IEMs, including MMA.	Fresh urine + 16% D_2O pH 2.5 adjusted with HCl and NaOH.<br>Urine dried on filter paper and stored in different conditions for different time intervals, was reconstituted in pure D_2O and recorded once again.<br><br>No reference compound mentioned.	One pulse (90° pulse, water presat during relaxation delay, 5 s recycling delay, 64 scans) at 298K.<br><br>One fresh urine spectrum and a dried paper reconstituted spectrum from another IEM are presented.		metabolites related to other IEMs. Citrate mentioned as easily degradable in time by bacteria and hippurate as a being a stable metabolite.		
Identified a previously unknown interreference in the routine MS analysis of dried blood spots (DBS)	Both DBS and urine. HPLC-NMR samples eluted with various mixtures of ultrapure water, acetonitrile and formic acid. For 2D NMR, HPLC evaporates have been reconstituted in 600 µL D2O.<br><br>No chemical shift reference compound mentioned.	500 MHz and 600 MHz (Bruker Avance III HD 500 and Avance Neo 600).<br><br>Hyphenated HPLC-NMR (HPLC-heart-cut-NMR) for identification of unknown compounds in urine. Spectra recorded with various NMR experiments including ^{1}H NMR, HSQC, HMBC and TOCSY using both ambient temperature probes and cryoprobes.	PA, MMA	MS interfering metabolites have been assigned by NMR to 2-methyl-3-hydroxybutyrate, 3-hydroxyisovalerate, 2-hydroxyisovalerate, 3-hydroxyvalerate and succinate. A sixth compound in low abundance has been also observed but its structure was not identified. Out of these isobaric compounds, only succinate has been previously reported to interfere with methylmalonic acid in DBS analysi	3 PA, 1 MMA	[72]
Quantified methylmalonic acid in a B_{12} deficient patient by ^{1}H NMR of cerebrospinal fluid (CSF)	Cerebrospinal fluid (CSF) sample was freeze dried and concentrated 5X by redissolving it in 85% H2O and 15% D_2O without addition of TSP,	300 MHz and 400 MHz (Varian XL300 and Varian VXR400S).<br>The CSF sample by one pulse spectrum of B12 deficiency recorded at 300 MHz (161 scans, 5 s acquisition time, 90° pulse and no relaxation delay).	B_{12} deficiency, control, other diseases.	Lactate, alanine, acetate, glutamine, citrate, creatine + creatinine, glucose, and methylmalonic acid assigned. Several other signals have been present in the NMR spectrum but they could not be assigned to particular metabolites. The methylmalonic acid concentration in CSF of the patient was estimated to 154 µM. Chemical shift	Control, a B_{12} deficiency, other diseases.	[73]

Key aspects	Sample preparation	NMR Experimental details	Disease	Mentioned metabolites / Features	Cohorts	Ref.
	resulting an acidic solution with pH 6.	The CSF one pulse spectrum was compared with control CSF recorded at 400 MHz (640 scans, 5 s acquisition time, 90° pulse, 12 s relaxation delay).<br>For signal assignment purposes COSY spectra have been recorded.<br>A CSF spectrum from a control, a B_{12} deficiency patient and other diseases are presented. COSY spectra are also presented.		dependency of pH for methylmalonic acid was also reported as titration curve.		
Cord blood revealed markers for potential risk of MMA and other two diseases in gestational hypothyroidism (GHT), which is a frequent pregnancy-related thyroid disfunction.	Cord blood. an aliquot of 450 µL plasma was thawed on ice and mixed with 900 µl methanol, vortexed for 2 min then centrifuged at 4 °C, 13,000 rpm for 20 min to pellet proteins. The supernatant was dried completely by a vacuum dryer. The dried sample was reconstituted in 600 µL phosphate buffer solution in D_2O (0.2 M pD=7.4, containing 10 mM TSP).	600. 600 MHz (Bruker AVANCE III).<br><br>CPMG spectra (1 s relaxation delay, 64 scans). The obtained NMR data was normalized to the total sum of spectra before further analysis.<br>Multivariate PCA and PLS-DA analysis.<br><br>CPMG spectrum with assignments presented.	GHT MMA, control	Cord blood metabolites with altered abundances between GHT and the NHT control reported as: L-alpha-aminobutyric acid, carnitine, citric acid, sarcosine, 3-methyl-2-oxovaleric acid, tyrosine, creatinine, O-phsphocholine. Several other metabolites assigned.	18 pregnant women with GHT and 18 non hypothyroidism (NHT) control	[74]
