# Supplementary material for: NMR Spectroscopy in Diagnosis and Monitoring of Methylmalonic and Propionic Acidemias
Source: Biomolecules. 2024 Apr 28;14(5):528. doi: 10.3390/biom14050528 (PMC11117674; doi:10.3390/biom14050528)
Supplement: Supplementary file 1 [file biomolecules-14-00528-s001.zip › Supplementary-Table-S2.pdf]

NMR spectroscopy in diagnosis and monitoring of methylmalonic and propionic acidemias

Calin Deleanu and Alina Nicolescu *

Supplementary 2

Table S2. Summary of localized MRS and MRI studies on MMA, PA and B_{12} deficiency.

Key aspects	NMR Experimental details	Disease	Mentioned metabolites / Features	Cohorts	Ref.
Several neurogenetic and metabolic disorders present non specific brain atrophy in MRI. Most of the MRI studies have been focusing on globus pallidus in basal ganglia, as they are metabolically very active and are influenced by metabolic abnormalities and toxic poisoning	Only MRI	Bilateral symmetrical basal ganglia and thalamic lesions in children, including MMA			[78] Review
Basal ganglia being affected more frequently in MMA than B_{12} deficiency.	1.5 Tesla (64 MHz proton frequency).<br><br>Only MRI	MMA, MMA-HC, B_{12} deficiency	Reduction in volume of white matter. Basal ganglia, and particularly the globi pallidi, were free of diffuse signal alterations in all cases.	14 cases	[79]
Assumed the reason for white matter bulk loss not being a consistent feature of late disease stages could reflect biochemical or genetic heterogeneity among affected patients. MR imaging features of MMA-HC at presentation are nonspecific and do not permit differentiation from other metabolic leukoencephalopathies; therefore, the diagnosis relies heavily on clinical and laboratory investigations.	Instrument not mentioned. Assumed 1.5 Tesla.<br><br>Only MRI	MMA, MMA-HC, B_{12} deficiency	Normal, moderate and severe reduction in volume of white matter.	16 cases	[80]
Basal ganglia being affected similarly in MMA and B_{12} deficiency.<br>Cerebrospinal fluid and brain metabolite concentrations do not always correlate with blood values, and this may explain why the	1.5 Tesla (64 MHz proton frequency).<br>SVS (with STEAM)	MMA, MMA-HC, B_{12} deficiency	Small pons, partial vermian dysgenesis, abnormal signal in both basal ganglia in MRI and low NAA in MRS of B12 deficiency; Normal MRI in a MMA-HC; Cysts in globus pallidus bilaterally in MRI and low NAA in MRS of a	18 controls 1 MMA, 1 MMA-HC, 1 B_{12} deficiency, 8 other	[81]

Key aspects	NMR Experimental details	Disease	Mentioned metabolites / Features	Cohorts	Ref.
MR studies were normal despite markedly raised metabolites in plasma.	An MRS spectrum of MMA-HC is presented.		MMA. All MMA, MMA-HC and B_{12} patients exhibited lower creatine and visible lactate peaks. Significant correlation between age and water content for both control and abnormal groups. There is a gradual drop of water content over the first few months of life, reaching a steady level by the age of 1 year. No significant difference in water content between controls and abnormal groups.	metabolic disorders	
Progressive MRI changes involving basal ganglia and white matter may occur in MMA, despite early initiation of B_{12}, the prevention of acute encephalopathic episodes, and improvement in measurable clinical biochemical parameters. Progression in biochemical improvement suggests a pathogenic mechanism based on cell autonomous effects of the failed cblC function.	Instrument not mentioned. Assumed 1.5 Tesla and SVS<br><br>MRI images presented.<br>No MRS spectrum presented.	MMA, B_{12} deficiency	Progressive MRI changes involving basal ganglia and white matter occurring in MMA, despite early initiation of B_{12}.	One case	[82]
Basal ganglia are affected similarly in MMA and B_{12} deficiency.<br><br>Basal ganglia lesions may be present in B12 deficiency (cbl-C/D defect), together with supratentorial white matter damage and tetraventricular hydrocephalus. This markedly heterogeneous neuroradiological picture does not correlate with biochemical and clinical findings.	1.5 Tesla (64 MHz proton frequency) (Magnetom Vision, Siemens).<br>CSI and SVS.<br><br>MRI and MRS spectra are presented.	B_{12} deficiency	In 3 out of 5 cases MRS showed lactate. In both patients with lesions in the basal ganglia, lactate was detected within these structures. In one patient lacking basal ganglia abnormalities, the lactate peak was localized in the periventricular white matter. For each patient the ratios of the integral values choline/creatine, NAA/creatine, NAA/choline were compared to the normal ratio values. Only one patient showed an elevated choline/creatine ratio and a diminished NAA/choline ratio which are compatible with myelination delay.	7 B_{12} deficiency patients	[83]
MRI detects abnormalities at a later stage of the disease. PA and MMA children shown only very subtle abnormalities during first months of life but they developed cerebral atrophy in the next year. During therapy, these changes often resolved, especially in the patients with methylmalonic acidemia.	1.5 Tesla (64 MHz proton frequency).<br><br>MRI presented.<br><br>No MRS	MMA, PA		20 PA and 23 MMA	[84]

Key aspects	NMR Experimental details	Disease	Mentioned metabolites / Features	Cohorts	Ref.
Findings were similar in the two syndromes. Children with MMA or PA, in addition to widening of cerebrospinal fluid spaces and some delay in myelination, often show symmetric involvement of the basal ganglia.”					
MRS could be a more specific and earlier diagnostic tool for these diseases than MRI.<br><br>Excellent introduction to MRI and MRS of brain.	Excellent introduction to MRI and MRS of brain.<br><br>Experimental details explained.	General			[38]
MRS could be a more specific and earlier diagnostic tool for metabolic diseases than MRI.	1.5 Tesla (64 MHz proton frequency)	General	Comparison of variability in-vivo and in-vitro for choline, NAA, and creatine.		[85]
MRS can easily monitor N-acetylaspartate (NAA) which is related to neurons’ activity, myoinositol (mI) which is thought to protect the brain by its osmolytic property, glutamate usually overlapped with glutamine (Glx), creatine usually overlapped with phosphocreatine (Cr+PCr), and choline usually overlapped with phosphocholine, glycerophosphorylcholine and other choline derivatives (Cho)	MRI and MRS spectra form normal and abnormal regions of the brain are presented for another IEMs.	MMA, PA, controls, other IEMs.	NNA concentration constantly increases from birth until about 20 years; myoinositol (mI) thought to protect the brain by its osmolytic property; glutamate usually overlapped with glutamine (Glx); creatine usually overlapped with phosphocreatine (Cr+PCr); choline usually overlapped with phosphocholine, glycerophosphorylcholine and other choline derivatives (Cho)		[50] Review
Brain MRS applications on several inborn and acquired metabolic diseases which are affecting brain metabolism, including MMA and PA	Good review.<br><br>No MRI and no MRS spectra are presented.	MMA, PA, other IEMs.			[39]
MRI sown in all PA patients delayed myelination and some cerebral atrophy. In one PA patient with choreoathetosis, MRI exhibited bilateral abnormalities in the putamen and caudate nuclei, while the others exhibited normal basal ganglia. MRS basal ganglia for all 3 PA decreased NAA, mI and increased glutamine/glutamate ratio. The study shown that MRS information from tissue (intracellular) could be different from the extracellularly one provided by urine, plasma and CSF, underlying that tissue and	1.5 Tesla (64 MHz proton frequency).<br><br>SVS (with STEAM).<br><br>Comparison of MRS spectra for a PA patient and a control are presented with assignments of NAA, Cr+PCr, mI and Glx.	PA	In all three patients decreased N-acetylaspartate (NAA) and myo-inositol (mI) and increased glutamine/glutamate ratio; assignments of N-acetylaspartate (NAA), creatine (Cr+PCr), choline (“choline-containing compounds”), myoinositol (mI) and Glx (glutamine, glutamate and GABA)	3 PA, 8 controls.	[86]

Key aspects	NMR Experimental details	Disease	Mentioned metabolites / Features	Cohorts	Ref.
body fluids provide complementary information.					
Compared MRI and MRS of basal ganglia for B_{12} deficiency, MMA, controls and other types of metabolic disorders. MRS spectra with metabolites assignments have been presented.	1.5 T (64 MHz proton frequency).<br><br>SVS (with STEAM) measured for each patient from the left or right basal ganglia.<br><br>MRI and MRS presented from a control, the B_{12} deficiency and other IEMs.	MMA, B_{12} deficiency, other IEMs, controls.	Levels of myoinositol, choline, creatine and NAA determined together with the water content of the measured region. NAA was lower in B_{12} deficiency and one of the two MMA patients.	1 B_{12} deficiency, 2 MMA, 17 controls, 10 other metabolic disorders	[81]
7 controls and 4 PA under treatment (with protein restriction and carnitine supplementation) have been studied by MRI and MRS. Studies have been done 3 up to 5 years after first diagnosis when no metabolic decompensation and no or minimal MRI abnormalities have been present.	1.5 T (64 MHz proton frequency) (Siemens Magnetom GBSII and Vision).<br><br>SVS (with STEAM) CSI.<br><br>MRI images and MRS spectra are presented.	PA, controls	MRS revealed lactate in all 4 PA while none of the controls had lactate. 2 PA patients diagnosed later, also exhibited decreased NAA/Cho ratios, while NAA/Cr and Cr/Cho ratios have been similar in both controls and PA.	7 controls and 4 PA under treatment (with carnitine).	[87]
The study highlighted the superiority of the newer multi-slice MRS and diffusion-weighted MRI over previous techniques as multi-slices may cover regions unsuspected by MRI abnormalities.<br>This study confirmed earlier observation that Lac concentration is not correlated in brain tissue (intracellular) and CSF (extracellular).	1.5 Tesla (64 MHz proton frequency).<br><br>CSI.<br><br>MRI, MRS and CSI-images are presented.	MMA	In one MMA in globus pallidus region increased Lac and decreased NAA have been observed, while Cr and Cho have been normal. In the other regions of the brain with no lesions, concentrations have been normal for NAA, Cr and Cho. In the second MMA, normal levels of NAA, Cr and Cho and no detectable Lac have been observed in all regions. However, Lac was high in all CSF spaces, especially in lateral ventricles.	2 MMA cases	[88]
Monitoring an MMA patient before and during therapy with carnitine and vitamin B_{12} was followed by MRI and MRS. MRI shown high signals in both basal ganglia. MRS during therapy revealed constant decrease of	1.5 Tesla (64 MHz proton frequency). (GE Signa Horizon).<br><br>SVS (with PRESS).	MMA	MRI shown high signals in both basal ganglia. MRS shown high lactate and decreased NAA in the same MRI. MRS recorded before initiation of the treatment and periodically up to 9 months during therapy revealed constant decrease of	One MMA before and during therapy with carnitine and vitamin B_{12}	[89]

Key aspects	NMR Experimental details	Disease	Mentioned metabolites / Features	Cohorts	Ref.
lactate until its complete disappearance and constant increase of the NAA level.	MRI and MRS are presented.		lactate until its complete disappearance and constant increase of the NAA level.		
A nutrition B_{12} deficiency in a breast-fed infant due to maternal malabsorption was studied by both MRI and MRS. The normalization of MRS spectrum was showing the reversibility of the condition when nutritional B_{12} deficiency is treated in early stages.	Instrument not mentioned. Assumed 1.5 Tesla.<br><br>SVS (with STEAM).<br><br>MRI and MRS are presented.	B_{12} deficiency	MRS was showing during the first examination high lactate in supratenorial gray and white matter. Low choline, inositol and normal levels of NAA and other metabolites have been observed in the white matter. Lower NAA, Cr, Cho, mI, Glu, Gln and higher Lac have been observed in the gray matter. During the second examination, six month later, after B_{12} therapy, although MRI was still showing intense signal in basal ganglia, in MRS all metabolites in both gray and white matter returned to normal values and lactate was no longer present. The normalization of MRS spectrum was matching the return to the normal of methylmalonic acid in urine and general improvement in all clinical, including mental, aspects.	One nutrition B_{12} deficiency in a breast-fed infant.	[90]
5 MMA with homocystinuria (MMA-HC) have been monitored by both MRI and MRS. In spite of the fact that all patients had high concentrations of methylmalonic acid in urine, the MRS spectra have been normal in both cases with normal MRI. The ratios choline/creatine, NAA/creatine and NAA/choline have been normal in all but one patient which shown increased choline/creatine and decreased NAA/choline.	1.5 Tesla (64 MHz proton frequency).<br><br>CSI and SVS.<br><br>MRI, MRS and CSI-image are presented.	MMA with homocystinuria (MMA-HC)	MRI in 3 patients shown in lesions in basal ganglia and one shown subependymal hemorrhage. MRS during chronic phase of the disease shown high lactate in basal ganglia in two cases, while in one case lactate was present in the periventricular white matter corresponding to the MRI lesions. The ratios choline/creatine, NAA/creatine and NAA/choline have been normal in all but one patient which shown increased choline/creatine and decreased NAA/choline.	5 MMA-HC being monitored.	[83]
MRS and MRI of 3 patients with succinyl-coenzyme A synthetase deficiency (SCS) combined with encephalomyopathy and mild MMA. The paper revealed that SUCLG1 mutation is associated with excretion of methylmalonic acid and C4-DC carnitine in urine and lesions of basal ganglia visible in MRI.	1.5 Tesla (64 MHz proton frequency) (GE Signa). MRS sampling method N/A. Assumed SVS.<br><br>MRI and MRS are presented.	Succinyl-coenzyme A synthetase deficiency (SCS) combined with encephalomyopathy and mild MMA	SUCLG1 mutation is associated with excretion of methylmalonic acid and C4-DC carnitine in urine and lesions of basal ganglia visible in MRI. All three patients shown MRI enhanced intensities in bilateral caudate nuclei and bilateral putamen. Lactate was present in basal ganglia of two patients.	3 SCS combined with encephalomyopathy and mild MMA.	[91]
An MRS study of eight children with PA with experiments performed both during	1.5 Tesla (64 MHz proton frequency) (Siemens	PA	Two children who had previously liver transplant did not show significant differences from the	8 PA during both metabolic stability	[92]

Key aspects	NMR Experimental details	Disease	Mentioned metabolites / Features	Cohorts	Ref.
metabolic stability and acute encephalopathic episodes. MRS quantitative data have been compared with a large control cohort, making this study a valuable reference for PA MRS. Published averaged spectra for metabolically stable PA versus acute encephalopathy episodes clearly underlines the power of MRS in differentiating these states. Two children who had previously liver transplant did not show significant differences from the control group, supporting thus the protective benefit of transplant.	Symphony Magnetom NUM4 and GE Signa Excite & HDx).<br><br>SVS (with PRESS).<br><br>MRS spectra presented.		control group. The paper describes significant decreases in basal ganglia of Glx (combined glutamine+glutamate) and N-acetylaspartate, together with increased lactate during encephalopathic episodes, whereas in the white matter only lactate was increased. During decompensations separate glutamine and N-acetylaspartylglutamate (tNAA) have been also decreased in basal ganglia. MRI during severe episodes shown abnormal signal basal ganglia, while the MRI was normal during metabolic stability.	and acute encephalopathic episodes.	
Nine patients with combined MMA and homocystinuria (MMA-HC) have been examined by MRI and MRS, they being under specific treatment during examination. All patients started treatment as early-onsets. The authors endorsed the previous assumption that plasma concentrations of MMA and homocysteine in early infancy are not well corelated with neurodevelopmental outcome.	Instrument not mentioned - assumed 1.5 Tesla.<br><br>MRS sampling method not mentioned - assumed SVS.<br><br>No MRI no MRS presented.	MMA and homocystinuria (MMA-HC)	Most frequent abnormalities have been listed as craniocaudally short pons, callosal thinning, and enhanced T2 FLAIR signal in periatrial and periventricular white matter. Basal ganglia MRI have been assessed as normal, as well as MRS spectra also assessed as normal in all patients, despite specific markers (methylmalonic acid, methionine, homocysteine and propionylcarnitine) in urine and plasma. Thus, guanidinoacetate was not seen and creatine was not decreased in MRS.	9 combined MMA and homocystinuria (MMA-HC).	[93]
A brain MRS spectrum of a MMA patient with methylmalonic acid assigned in the spectrum. One of the rare spectra where the authors, apart from lactate, identified in MRS the methylmalonic acid signal.	1.5 Tesla (64 MHz proton frequency).<br><br>SVS (with PRESS).<br><br>MRI and MRS presented.	MMA	Methylmalonic acid assigned in the spectrum	One MMA patient	[36] Book
MRI and MRS in 14 patients with nutritional vitamin B_{12} deficiency. Pre- and post-treatment MRI and MRS images and spectra are presented	3 Tesla (127.7 MHz proton frequency) (Philips Achieva).<br>SVS (with PRESS).<br><br>MRI and MRS are presented.	B_{12} deficiency	Eight cases shown abnormal MRI, most common issues being thinning of the corpus callosum and brain atrophy. In MRS lactate was not present in any patient and ratios NAA/Cr and Cho/Cr have been assessed as normal after treatment. Values of brain NAA/Cr and Cho/Cr ratios together with vitamin B_{12} concentrations in blood are reported.	14 patients with nutritional vitamin B_{12} deficiency.	[94]

Key aspects	NMR Experimental details	Disease	Mentioned metabolites / Features	Cohorts	Ref.
A study aiming to establish the cause of several non-compressive myelopathies in a tertiary care hospital from India. Thus, 55 patients with non-compressive myelopathies, have been assigned after MRI of the spine as chronic myelopathy (CM). In four of these patients the cause of CM has been identified as vitamin B_{12} deficiency. All B_{12} patients had a good outcome after metabolic supplementation.	1.5 Tesla (64 MHz proton frequency) (Siemens Magnetom).<br><br>Although MRS was mentioned as performed analysis, no details have been given and only MRI details and images have been presented.	B_{12} deficiency	Spinal cord MRI was performed on all patients and brain MRI was performed on selected patients. The level of vitamin B_{12} in serum was also analyzed in all patients.	55 patients with chronic myelopathy (CM). 4 of these patients identified as vitamin B_{12} deficiency.	[95]
Combined methylmalonic and homocystinuria (MMA-HC) patients and controls have been examined by both MRI and MRS. All patients have been examined by MRI and MRS before starting the treatment. Patients showed different MRI patterns of abnormalities, main features being hydrocephalus and supratentorial white matter edema. None of the patients exhibited abnormal MRI signals in the basal ganglia, and this was mentioned by the authors as a distinctive feature of MMA-HC in comparison with isolated MMA and PA.	1.5 Tesla (64 MHz proton frequency) (GE Signa).<br>SVS (with PRESS).<br><br>MRI and MRS are presented.	Methylmalonic and homocystinuria (MMA-HC), Controls	Main MRI abnormalities have been hydrocephalus and supratentorial white matter edema. None of the patients exhibited abnormal MRI signals in the basal ganglia. Three patients with no lesions in the basal ganglia exhibited Lac signal in MRS of these regions. The ratios NAA/Cr and NAA/Cho have been decreased in all patients and lactate was present in three patients. No abnormal values have been noticed for ratios Cho/Cr and mI/Cr. Moreover, it was shown that there was no Cho increases in patients, thus the reductions of NAA/Cho ratio being solely generated by decrease of NAA. There have been no correlations between NAA/Cr values and any biochemical markers in blood.	28 combined methylmalonic and homocystinuria (MMA-HC) and 21 controls.	[96]
Eighteen B_{12} deficiency patients without developmental delays and 12 controls have been examined by MRI and MRS from three different regions of the brain in order to evaluate levels of brain metabolites. The authors indicated that their result is similar with a nutritional study when healthy voluntaries have been administrated high doses of B-vitamins and no metabolic differences have been observed between supplemented and placebo groups. In spite of this conclusion, the authors reported that for	1.5 Tesla (64 MHz proton frequency) (Siemens Magnetom Symphony)<br><br>SVS (with PRESS)<br><br>MRI are presented. No MRS spectrum is presented.	B_{12} deficiency, Controls	MRI and MRS recorded from three different regions of the brain in order to evaluate levels of brain metabolites. MRI was revealing periventricular white matter hyperintensities in two patients, and brain atrophy in four patients. Lactate was not presented in any of the subjects. Ratios of NAA/Cr, Cho/Cr, mI/Cr, and Glx/Cr have been evaluated for all participants and the authors reported that there have been no differences in any of the metabolite ratios between B_{12} deficiency patients and controls. Concentration ratios for these metabolites in all	18 B_{12} deficiency patients without developmental delays and 12 controls.	[97]

Key aspects	NMR Experimental details	Disease	Mentioned metabolites / Features	Cohorts	Ref.
the only two patients who have been examined by MRS before and after starting the treatment, the ratios of NAA/Cr, Cho/Cr and mI/Cr have been increased while Glx/Cr decreased.			three studied regions for both pathological and control groups are presented. For the only two patients who have been examined by MRS before and after starting the treatment, the ratios of NAA/Cr, Cho/Cr and mI/Cr have been increased while Glx/Cr decreased.		
A cohort of 35 patients with vitamin B_{12} deficiency have been followed by MRI and MRS (only 24 by MRS). There was no control group in the study. 26 patients had abnormal MRI. Most of the patients' mothers have been reported as vegetarian. All patients had neurologic symptoms as well as low serum vitamin B_{12} concentrations.<br>No correlation between MRS metabolites ratios with motor/mental development and blood levels of vitamin B_{12} and homocysteine have been found, although in 74% of cases neuroimaging abnormalities have been identified.	1.5 Tesla (64 MHz proton frequency) (Philips Achieva).<br>SVS (with PRESS).<br><br>MRI are presented. No MRS spectrum is presented.	B_{12} deficiency	26 patients had abnormal MRI. All patients had neurologic symptoms as well as low serum vitamin B_{12}. Ratios of Cho/Cr and NAA/Cr have been measured by MRS and their values are reported. Graphic correlations of MRS metabolites ratios with motor/mental development as well as their correlations with blood levels of vitamin B_{12} and homocysteine are presented. No correlation between these parameters have been found although in 74% of cases neuroimaging abnormalities have been identified.	35 patients with vitamin B_{12} deficiency followed by MRI and MRS (only 24 by MRS).	[99]
